# Lysine Acetyltransferase 6A Drives M1 Macrophage Polarization Through Metabolic Reprogramming in Sepsis-Induced Acute Lung Injury

**DOI:** 10.3390/biom16040609

**Published:** 2026-04-20

**Authors:** Xin Wang, Junlin Chen, Yimei Lai, Yumeng Wang, Kaixia Hu, Mengshi Wu, Niansheng Yang, Yuefang Huang

**Affiliations:** 1 Department of Pediatrics, The First Affiliated Hospitalof Sun Yat-sen University, 58 Zhongshan Second Road, Guangzhou 510080, China; wangx866@mail2.sysu.edu.cn (X.W.); chenjlin29@alumni.sysu.edu.cn (J.C.); hukx8@mail2.sysu.edu.cn (K.H.); wumsh23@mail2.sysu.edu.cn (M.W.); 2 Department of Rheumatology and Clinical Immunology, The First Affiliated Hospital of Sun Yat-sen University, 58 Zhongshan Second Road, Guangzhou 510080, China; laiym8@mail.sysu.edu.cn (Y.L.); wangym89@mail2.sysu.edu.cn (Y.W.); yangnsh@mail.sysu.edu.cn (N.Y.)

**Keywords:** KAT6A, M1 macrophage, metabolic reprogramming, PI3K-AKT-mTOR signaling, sepsis, acute lung injury

## Abstract

Macrophage-mediated inflammation is a key driver of sepsis-induced acute lung injury (ALI). M1 macrophage polarization relies on metabolic reprogramming, yet the upstream regulatory factors remain unclear. Lysine acetyltransferase 6A (KAT6A), a MYST-family acetyltransferase, regulates transcriptional programs in immune cells, but its role in macrophage function and ALI progression remains unknown. Public single-cell and bulk transcriptomic datasets were used to assess KAT6A expression changes and its association with inflammatory and metabolic pathways in macrophages. KAT6A inhibition with WM1119 was used to evaluate effects on M1 polarization, cytokine production, metabolic reprogramming, and PI3K-AKT-mTOR signaling. The therapeutic potential of KAT6A inhibition was validated in a cecal ligation and puncture (CLP)-induced sepsis model by assessing lung injury, bacterial clearance, and survival. KAT6A expression was upregulated in sepsis and particularly enriched in M1 macrophages. Inhibition of KAT6A reduced inflammatory and glycolytic transcriptional programs, suppressed glycolysis and enhanced oxidative phosphorylation, leading to decreased cytokine production and limited M1 polarization accompanied by suppression of PI3K-AKT-mTOR pathway. In CLP-induced septic mice, treatment with the KAT6A inhibitor WM1119 alleviated lung injury, improved bacterial clearance, and prolonged survival. KAT6A expression is associated with macrophage glucose metabolism, pro-inflammatory responses, and M1 macrophage polarization in sepsis-induced acute lung injury. Pharmacologic inhibition of KAT6A may provide a promising therapeutic strategy for reducing macrophage-driven lung injury.

## 1. Introduction

Sepsis is a life-threatening syndrome marked by dysregulated host immunity, leading to systemic inflammation and multiple organ dysfunction [1,2]. The lung is the most susceptible organ, with up to 50% of septic patients developing acute lung injury (ALI) [3,4], conditions frequently progressing to respiratory failure with mortality rates as high as 60% [5]. The pathogenesis of sepsis-induced ALI remains poorly understood, partly due to limited knowledge of how inflammatory responses and immune-cell metabolic reprogramming drive lung injury during sepsis. A clearer understanding of these processes is needed to identify upstream regulators and potential therapeutic targets.

Immune cells are key drivers of ALI pathogenesis, with macrophages occupying a central role. Macrophages, as a dominant component of innate immunity, play roles in pathogens’ phagocytosis, antigen delivery, and inflammatory cytokines release in the surveillance against septic infection, promoting inflammation responses [6]. During the early phase of infection, macrophages predominantly polarize toward the M1 state, producing inflammatory mediators that facilitate pathogen elimination and activate adaptive immunity [7]. However, excessive or uncontrolled M1 activation precipitates cytokine storm and accelerates the progression of severe sepsis [8]. These observations have highlighted M1 polarization as a potential therapeutic target in sepsis-induced ALI.

Metabolic reprogramming has emerged as a central regulator of macrophage activation and polarization [9,10]. During inflammatory stimulation, macrophages undergo a rapid shift toward glycolysis, which sustains the bioenergetic and biosynthetic demands of M1 polarization and amplifies pro-inflammatory cytokine production [11]. Although several signaling pathways have been implicated in promoting glycolytic flux [12,13,14], the upstream transcriptional and chromatin-associated mechanisms that initiate and maintain this metabolic state in sepsis remain poorly defined. Moreover, how these metabolic shifts are integrated with inflammatory signaling to drive macrophage-mediated lung injury is not fully understood. Therefore, identifying upstream regulators that govern macrophage metabolic reprogramming and drive inflammatory phenotypes is essential for elucidating the pathogenesis of sepsis-induced ALI.

Epigenetic regulation represents a fundamental determinant of macrophage activation and polarization in sepsis [15,16]. As a major epigenetic mechanism, histone acetylation dynamically shapes transcriptional programs that influence both inflammatory responses and metabolic pathways, and is tightly controlled by the opposing activities of histone acetyltransferases (HATs) and deacetylases (HDACs) [17]. Among these enzymes, lysine acetyltransferase 6A (KAT6A), a member of the MYST family of HATs, was originally identified through recurrent chromosomal translocations in acute myeloid leukemia and is now recognized to regulate diverse cellular pathways and functions [18,19,20,21]. More recently, KAT6A has been implicated in promoting proinflammatory gene expression in activated macrophages [22,23], and KAT6A deficiency has been shown to attenuate macrophage-driven inflammatory responses in a periodontitis model [24]. Moreover, KAT6A loss has been shown to reduce H3K27 acetylation at NF-κB binding sites on inflammatory gene promoters in macrophages [24], directly linking its acetyltransferase activity to pro-inflammatory transcription. Together, these observations suggest that KAT6A may serve as an important epigenetic regulator of inflammatory macrophage programs. Nevertheless, whether KAT6A also regulates the metabolic reprogramming that supports M1 polarization, and how it contributes to sepsis-induced ALI, remains largely unexplored.

In this study, we found that KAT6A is upregulated in macrophages under inflammatory conditions both in vitro and in vivo. Functionally, KAT6A inhibition was associated with suppressed glycolysis and enhanced oxidative phosphorylation, accompanied by reduced inflammatory cytokine production and M1 polarization. In a cecal ligation and puncture (CLP)-induced sepsis model, pharmacological inhibition of KAT6A reduced inflammatory cytokine production, limited M1 polarization, alleviated acute lung injury, and improved survival. These findings support a role for KAT6A in macrophage metabolic and inflammatory programming during sepsis and suggest that KAT6A inhibition may offer therapeutic potential in sepsis-induced ALI.

## 2. Materials and Methods

### 2.1. Animals

C57BL/6J mice aged 8–10 weeks were obtained from Guangdong Yaokang Biotechnology (Guangzhou, China). Animals were maintained under specific pathogen-free (SPF) conditions in the Experimental Animal Center of Sun Yat-sen University. The mouse experimental protocol was approved by the Ethics Committee of Sun Yat-sen University, and all experiments were performed in accordance with the National Institutes of Health Guide for Care and Use of Animals.

### 2.2. Cecal Ligation and Puncture (CLP) Model

Polymicrobial sepsis was induced in C57BL/6J mice through cecal ligation and puncture, following an established method [25]. Under anesthesia, mice were placed on a sterile surgical field, and a 1-cm midline laparotomy was performed to access the peritoneal cavity. The cecum was isolated, and approximately 50% of its length distal to the ileocecal valve was ligated using 3-0 silk suture. Two perforations were made in the ligated segment with a 22-gauge needle, and a minimal amount of fecal content was extruded to facilitate bacterial dissemination. The cecum was repositioned into the abdominal cavity, and the incision was closed in two layers. Each mouse received 1 mL of pre-warmed sterile saline via subcutaneous injection for fluid resuscitation. Sham-operated animals underwent identical surgical exposure without ligation or perforation. All animals were sacrificed 12 h post-procedure.

### 2.3. Histological Assessment

Lung, colon, and liver tissues were flushed with PBS and fixed overnight in 4% neutral paraformaldehyde, followed by paraffin embedding. Sections (4 μm) were deparaffinized through graded alcohols, stained with hematoxylin and eosin (H&E), and examined under bright-field microscopy (Olympus, Center Valley, PA, USA).

Lung injury was scored according to the presence of hyperemia, hemorrhage, neutrophil infiltration in the airspace or vessel wall, and alveolar wall thickening or hyaline membrane formation. Each parameter was graded on a semiquantitative scale from 0 (no injury) to 4+ (maximal injury, >75% of the lung field) [26]. Liver necrosis was assessed using a five-point scale: 0, no necrosis; 1, single-cell necrosis; 2, ≤30% necrosis; 3, 31–60% necrosis; and 4, >60% necrosis [27]. Colon injury was evaluated based on four parameters, including mucosal bleeding ulcers, interstitial edema, inflammatory cell infiltration, and disruption of glandular architecture. Each parameter was scored from 0 (normal) to 4 (≥75% affected area); the mean value of the four component scores was used as the overall colon injury score [28]. All histological evaluations were performed independently by two pathologists in a blinded manner.

### 2.4. Immunofluorescence Staining

For immunofluorescence staining, bone marrow-derived macrophages (BMDMs) were fixed, permeabilized, and blocked with 3% bovine serum albumin (BSA, asegene, Guangzhou, China, Cat# 43035-100). The cells were then incubated overnight at 4 °C with primary antibodies against KAT6A (rabbit anti-human/mouse IgG, HUABIO, Hangzhou, China, 1:500, Cat# HA723157) or F4/80 (rat anti-human/mouse IgG, 1:100, Invitrogen, Carlsbad, CA, USA, Cat#2739556). After washing with PBS, cells were incubated with Alexa Fluor 488-conjugated rabbit anti-rat secondary antibody (Invitrogen, Cat# A21210) or Alexa Fluor 594-conjugated goat anti-rabbit secondary antibody (Invitrogen, Cat# A11012) at room temperature (RT) for 1 h, followed by DAPI (SouthernBiotech, Birmingham, AL, USA, Cat# 0100-20) staining. Images were acquired using a fluorescence microscope (Olympus).

### 2.5. Single-Cell RNA Sequencing (scRNA-Seq) Analysis

Mouse lung scRNA-seq data were obtained from the GEO database (https://www.ncbi.nlm.nih.gov/geo/, accessed on 11 July 2022) with the accession number GSE207651, comprising samples from both sham-operated and CLP-induced septic mice. Data preprocessing was conducted using the Seurat package (v 5.4.0) to generate gene expression matrices. Quality control criteria included retaining cells with a minimum of 200 and a maximum of 6000 detected genes per cell, and excluding cells with mitochondrial transcript content exceeding 20%; genes expressed in fewer than 3 cells were also excluded from analysis. Uniform Manifold Approximation and Projection (UMAP) was applied for nonlinear dimensionality reduction to visualize transcriptional heterogeneity. Cluster identification was performed by integrating canonical marker genes from the CellMarker and PanglaoDB databases with annotations derived from relevant literature. Data analysis and visualization were conducted in R (v4.4.1) using RStudio.

### 2.6. Isolation, Culture, and Treatment of Macrophages

BMDMs were isolated using established procedures [29]. Mice were euthanized by rapid cervical dislocation, and femurs along with tibias were aseptically collected from the hind limbs. After trimming the bone ends near the joints, marrow cells were flushed out using BMDM differentiation medium. The resulting suspension was filtered through a 70 μm strainer and centrifuged at 500× *g* for 5 min at 4 °C. Bone marrow cells were cultured in DMEM (Gibco, Thermo Fisher Scientific, Waltham, MA, USA, Cat# C11995) containing 20% fetal bovine serum (FBS, Procell, Wuhan, China, Cat# 164210) and 1% penicillin/streptomycin (PS, Gibco, Cat# 15140122) at 37 °C in a humidified atmosphere with 5% CO_2_. M0 macrophages were induced by supplementing the cultures with 20 ng/mL macrophage colony-stimulating factor (M-CSF, PeproTech, Cranbury, NJ, USA, Cat# 315-02-10) for 6 days. For KAT6A inhibition, BMDMs were pretreated with WM1119 (MedChemExpress, Monmouth Junction, NJ, USA, Catalog# HY-102058) at the indicated concentrations for 1 h prior to lipopolysaccharide (LPS, 100 ng/mL, MilliporeSigma, Burlington, MA, USA, Cat# L2880) stimulation.

To obtain thioglycolate-elicited peritoneal macrophages (PMs) [30], mice were administered an intraperitoneal injection of 3 mL 4% thioglycolate broth. After 72 h, 5 mL PBS was injected into the peritoneal cavity using a 25 G needle, and the abdomen was gently massaged to dislodge cells. The lavage fluid was collected, centrifuged at 500× *g* for 10 min at 4 °C, and the cell pellet was resuspended in RPMI 1640 (Gibco, Cat# C11875) containing 10% FBS and 1% PS. Cells were incubated at 37 °C in a humidified atmosphere with 5% CO_2_. Cells were plated and allowed to adhere for 6 h, after which non-adherent cells were removed by washing with PBS. For KAT6A inhibition, PMs were pretreated with WM1119 at the indicated concentrations for 1 h prior to LPS (100 ng/mL) stimulation.

For M1 differentiation [31], macrophages were stimulated with 100 ng/mL LPS and 50 ng/mL recombinant mouse interferon-gamma (IFN-γ, Peprotech, Cranbury, NJ, USA, Cat# 315-05-20). For KAT6A inhibition [32], WM1119 was added 1 h before LPS and IFN-γ treatment. The cells were harvested 48 h later, and M1 polarization was assessed by flow cytometry and quantitative real-time PCR (qPCR).

### 2.7. Bulk RNA-Seq

PMs from C57BL/6J mice were stimulated with LPS in the presence or absence of 100 μM WM1119 for 6 h. RNA libraries were prepared and sequenced on the BGIseq500 platform (BGI) to generate 150 bp paired-end reads. HISAT2 (v2.2.1) was used for alignment to the mouse reference genome (GRCm38), and gene-level counts were obtained with featureCounts (v2.0.1). Genes with >10 total reads across all samples were retained. Batch effects were removed with the ComBat-seq function in the sva package (v3.59.0). The limma method was applied to identify DEGs [33]. The cutoff values of statistical significance were set at a *p*-value < 0.05 and a |log_2_ (fold change)| > 0.585. Kyoto Encyclopedia of Genes and Genomes (KEGG) pathway enrichment analysis was performed using the clusterProfiler R package (v4.1.0) [34]. Gene Set Enrichment Analysis (GSEA) against the MSigDB hallmark gene sets, and subsequent leading-edge analysis were conducted using the clusterProfiler (v 3.2.9). Significantly enriched hallmark pathways (*p* < 0.05) were visualized and ranked by their normalized enrichment score (NES).

### 2.8. Real-Time Quantitative PCR (qPCR)

Total RNA was isolated using TRIzol reagent (Invitrogen, Cat# 15596018) and reverse-transcribed into cDNA with Evo M-MLV RT Premix kit (Accurate Biotechnology, Changsha, China, Cat# AG11706). SYBR Green-based qPCR (Accurate Biotechnology, Cat# AG11718) was performed under the following conditions: 95 °C for 30 s, then 40 cycles of 95 °C for 5 s and 60 °C for 30 s. β-actin served as the internal control. Primer sequences are listed in Supplementary Table S1.

### 2.9. Glucose Uptake Assay

As previously described [35], glucose uptake was determined using the fluorescent analog 2-NBDG (Thermo Fisher Scientific, Cat# N13195). BMDMs were incubated in glucose-free DMEM containing 50 μM 2-NBDG for 30 min at 37 °C, and fluorescence intensity was quantified by flow cytometry.

### 2.10. Flow Cytometry

For surface marker analysis, single-cell suspensions were stained with the following antibodies: Pacific Blue anti-mouse F4/80 (Cat# 123124), APC/Cyanine7 anti-mouse CD86 (Cat# 105030), Brilliant Violet 510 anti-mouse CD45.2 (Cat# 109838), PerCP/Cyanine5.5 anti-mouse CD11c (Cat# 117328), PE/Cyanine7 anti-mouse CD3 (Cat# 100220), PE anti-mouse CD19 (Cat# 152408), PE/Dazzle 594 anti-mouse I-A/I-E (Cat# 107648), Alexa Fluor 647 anti-mouse CD170 (Cat# 155519), Alexa Fluor 700 anti-mouse/human CD11b (Cat# 101222), APC/Cyanine7 anti-mouse Ly6G (Cat# 127624) at 4 °C for 30 min. For intracellular cytokine analysis, cells were stimulated with 5 μg/mL Brefeldin A (BFA, Sigma-Aldrich, Cat# B7651) at 37 °C in a humidified atmosphere with 5% CO_2_ for 4 h. Cells were initially treated with the Fixation/Permeabilization Kit (BD Biosciences, Franklin Lakes, NJ, USA, Cat# 554722), then stained with PE anti-mouse IL-6 (Cat# 504503), PerCP/Cyanine5.5 anti-NOS2 (Cat# 696809) and PerCP/Cyanine5.5 anti-mouse TNFα (Cat# 506321). To measure the expression of p-AKT or p-S6, cells were stained with antibodies against p-AKT^S473^ (Cell Signaling Technology, Danvers, MA, USA, Cat# 4060S) or p-S6^S235/236^ (Cell Signaling Technology, Cat# 4858S), followed by goat anti-rabbit IgG (Thermo Fisher Scientific). Cells were labelled with Annexin V-APC and PI (MultiSciences, Hangzhou, China, Cat# AP101PI-100) to assess cell viability by flow cytometry. We purchased all antibodies from Biolegend (San Diego, CA, USA) unless specified otherwise. Data were acquired using an LSR Fortessa flow cytometer (BD Biosciences) and analyzed with consistent gating strategies.

### 2.11. Western Blot

BMDMs and murine lung tissues were homogenized and lysed in radioimmunoprecipitation assay (asegene, Guangzhou, China, Cat# LB018A) buffer supplemented with protease inhibitors (MCE, Shanghai, China, Cat# HY-K0010) and phosphatase inhibitors (asegene, Cat# P004861). The total protein content of each lysate was quantified using the bicinchoninic acid (BCA) assay (Epizyme Biomedical Technology, Shanghai, China, Cat# ZJ102). Equal amounts of protein were resolved on 10% SDS–polyacrylamide gels and subsequently transferred to polyvinylidene difluoride (PVDF) membranes (Millipore, Bedford, MA, USA, Cat# ISEQ00010). Membranes were blocked with 5% BSA in Tris-buffered saline containing 0.1% Tween-20 (Aladdin, Shanghai, China, Cat# T104863) and then incubated overnight at 4 °C with primary antibodies specific for Pyruvate Kinase M2 (PKM2, 1:1000, Cell Signaling Technology, Cat# 4053S), p-PI3K^p85^ (1:1000, Cell Signaling Technology, Cat# 17366S), p-AKT^S473^ (1:2000, Cell Signaling Technology, Cat# 4060S), PI3K (1:1000, Cell Signaling Technology, Cat# 4257T), AKT(1:1000, Cell Signaling Technology, Cat# 4691T), p-mTOR^S2448^ (1:1000, Cell Signaling Technology, Cat# 5536T), mTOR (1:1000, Cell Signaling Technology, Cat# 2983T), Raptor (1:1000, Cell Signaling Technology, Cat# 2280T), hypoxia-inducible factor 1-alpha (HIF1α, 1:1000, Abmart, Shanghai, China, Cat# T55824F), glucose transporter 1 (GLUT1, 1:500, Abmart, Cat# T55360F), Cellular Myc (c-Myc, 1:500, Abmart, Cat# T55150F), H3K9ac (1:1000, Cell Signaling Technology, Cat# 9649T), H3K27ac (1:1000, Cell Signaling Technology, Cat# 8173T), Histone H3 (1:2000, Cell Signaling Technology, Cat# 4499T) and β-actin (1:1000, Servicebio, Wuhan, China, Cat# GB15003). Membranes were washed with TBST, incubated with horseradish peroxidase-labelled anti-rabbit (1:10,000, Servicebio, Wuhan, China, Cat# GB23303) for 1 h at RT, and developed using a chemiluminescence detection kit (EpiZyme, Shanghai, China, Cat# SQ201L).

### 2.12. Cell Transfection

Small interfering RNA targeting KAT6A (siKAT6A) was synthesized by Sangon Biotech (Shanghai, China). The sense and antisense sequences of siKAT6A were 5′-GACGGAAACUUCUACAAAAdTdT-3′ and 5′-UUUUGUAGAAGUUUCCGUCdTdT-3′, respectively. For transfection, RAW264.7 cells in the logarithmic growth phase were seeded into 24-well plates to achieve 70–80% confluence. Cells were then transiently transfected with either scramble or KAT6A siRNA using Lipofectamine 2000 reagent (Invitrogen, Cat# 11668) according to the manufacturer’s instructions. Briefly, siRNA and Lipofectamine 2000 were separately diluted in Opti-MEM medium (Gibco, Cat# 31985070), then mixed at a 1:1 ratio and incubated for 5 min at room temperature to form siRNA-lipid complexes. The resulting mixtures were added to the culture wells. After 48 h of incubation, the cells were harvested for subsequent experimental analysis.

### 2.13. Measurement of Lactate Content

The lactate concentrations in the samples were determined using a colorimetric Lactate Content Assay Kit (Solarbio, Beijing, China, Cat# BC2235) according to the manufacturer’s protocol. For the pharmacological experiments, BMDMs were stimulated with LPS and treated with either the KAT6A inhibitor WM1119 or DMSO. For the gene knockdown experiments, RAW264.7 cells were transfected with scramble or KAT6A siRNA followed by LPS stimulation. Briefly, cells were processed and centrifuged at 12,000× *g* for 10 min at 4 °C to collect the supernatant. The absorbance was measured at 570 nm using a microplate reader. The lactate content was calculated based on the ratio of the sample absorbance to the standard absorbance using the formula provided by the manufacturer.

### 2.14. Seahorse Extracellular Flux Assay

Immunometabolic profiling of macrophages was performed by Seahorse metabolic flux analysis using the Cell Mito Stress Test or Glycolysis Stress Test kits (Agilent Technologies, Santa Clara, CA, USA), as previously described [36]. For macrophage assays, BMDMs were seeded at 0.1 million cells per well in Seahorse XF96 culture plates (Agilent Technologies). Mitochondrial respiration was evaluated through sequential injections of oligomycin (1.5 μM), carbonyl cyanide-p-trifluoromethoxyphenylhydrazone (FCCP, 1.5 μM), and a combination of rotenone/antimycin A (1.0 μM). Glycolytic function was determined by sequential administration of glucose (10 mM), oligomycin (1 μM), and 2-deoxy-D-glucose (2-DG, 50 mM). Data were collected with Agilent Seahorse XFe96 and analyzed by Wave software version 2.6.4 (Agilent Technologies).

### 2.15. Serum Enzyme

Blood samples were collected from mice 24 h after surgery and allowed to clot at RT for 5 h. Samples were then centrifuged at 3000 rpm for 10 min to obtain serum, which was stored at −80 °C until analysis. Serum levels of aspartate aminotransferase (AST), alanine aminotransferase (ALT), creatine kinase (CK), urea, and creatinine (Crea) were measured using an automatic biochemical analyzer (Mindray BS-240VET, Shenzhen, China).

### 2.16. Bacteria Culture

Peritoneal lavage fluid was collected 24 h after CLP induction by irrigating the peritoneal cavity with 3 mL of sterile PBS. For assessment of bacterial load in the lung, fresh tissue was weighed and homogenized in 1 mL of PBS. Aliquots (100 μL) of either peritoneal fluid or lung homogenates were thoroughly mixed and subjected to serial 10-fold dilutions in PBS. The diluted samples were then plated onto Luria-Bertani (LB, Sangon Biotech, Shanghai, China, Cat# A507002) agar plates and incubated overnight at 37 °C, after which bacterial colonies were enumerated.

### 2.17. Statistical Analysis

Results are expressed as mean ± SEM. Sample sizes were selected based on prior in vivo sepsis studies using the CLP model and published literature, with group sizes of n = 3–10 per group as specified in individual figure legends. Statistical significance was determined using two-tailed unpaired Student’s *t*-test for two-group comparisons or one-way ANOVA with post hoc correction for multiple comparisons, as appropriate, in GraphPad Prism v10.1.2. Survival analysis was performed using the Kaplan–Meier method. A *p*-value < 0.05 was considered statistically significant.

## 3. Results

### 3.1. KAT6A Is Upregulated in Macrophages During Sepsis

**Figure 1:**
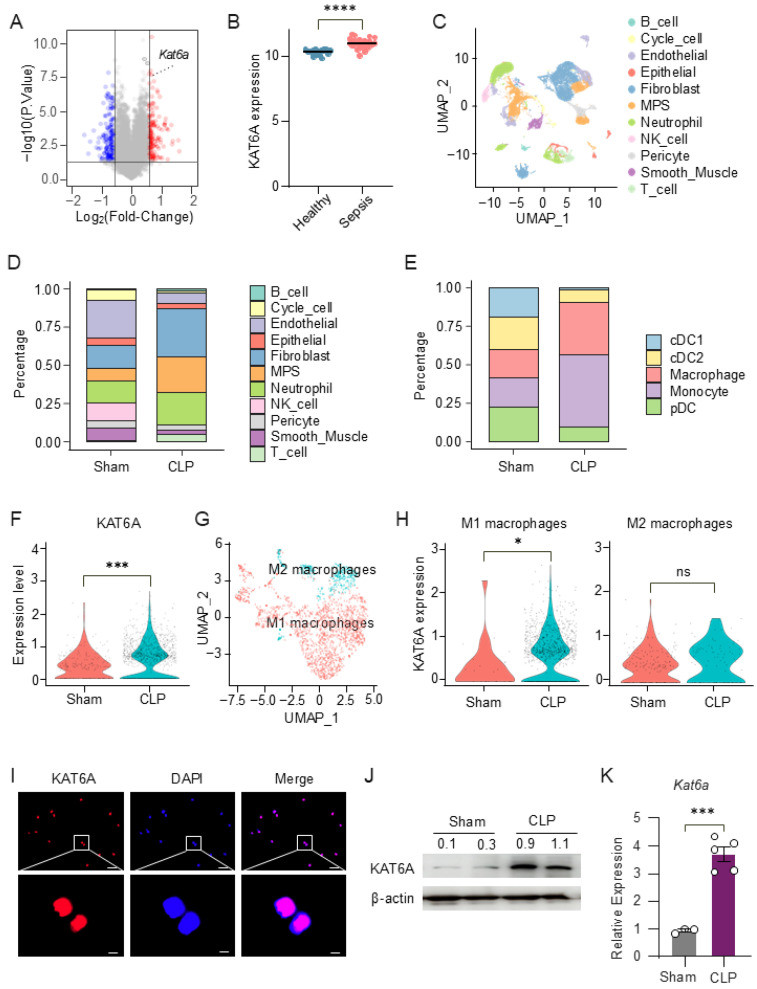
Lysine acetyltransferase 6A (KAT6A) is upregulated in macrophages during sepsis. (**A**) Microarray-based transcriptomic analysis of peripheral blood samples from healthy individuals and sepsis patients in the GSE54514 dataset. Upregulated genes (red), downregulated genes (blue), and stable genes (grey) are indicated. (**B**) Expression levels of KAT6A in healthy individuals (n = 18) and sepsis patients (n = 26). (**C**–**H**) Single-cell RNA sequencing analysis of lung tissues from sham and CLP mice (GSE207651). (**C**) Uniform Manifold Approximation and Projection (UMAP) visualization of cells from sham and CLP groups. (**D**) Bar chart depicting the relative abundances of different cell populations. (**E**) Bar chart of the relative abundances of cells in the mononuclear phagocyte (MPS) clusters. (**F**) Violin plot illustrates the distribution of KAT6A expression in macrophages from sham and CLP mice. (**G**) UMAP visualization of M1 and M2 macrophage subpopulations identified within the macrophage cluster. (**H**) Violin plots of KAT6A expression in M1 and M2 macrophage subsets. (**I**) Representative confocal microscopy images of KAT6A (red) and DAPI (blue) in peritoneal macrophages (PMs). scale bar: 20 µm (top); scale bar, 2 µm (bottom). (**J**) KAT6A expression in lung tissues from septic and sham-operated mice was analyzed by Western blot. The experiment was repeated three times independently with similar results. (**K**) Quantitative real-time PCR (qPCR) analysis of KAT6A mRNA levels in lung tissues from sepsis mice (n = 5) and sham-operated mice (n = 3). All data are mean ± SEM. * *p* < 0.05, *** *p* < 0.001, **** *p* < 0.0001 by two-tailed unpaired Student’s *t* test in panel (**K**). ns, not significant. Original Western blot images can be found in Figure S9.

To determine whether lysine acetyltransferase 6A (KAT6A) expression is altered in sepsis patients, we analyzed microarray data from the public dataset GSE54514. Differential expression analysis identified 113 significantly upregulated and 129 downregulated genes in sepsis patients compared with healthy individuals, with *Kat6a* among the significantly upregulated transcripts (Figure 1A). Furthermore, KAT6A expression was also significantly elevated at the individual gene level (Figure 1B), suggesting a potential involvement of KAT6A in the host immune response during sepsis.

To further investigate KAT6A expression at the single-cell level, we analyzed a publicly available single-cell RNA-seq dataset of lung tissues from cecal ligation and puncture (CLP)-induced septic mice or sham controls (GSE207651). Unsupervised UMAP clustering identified distinct immune and stromal cell populations in sham and CLP-treated mice based on transcriptional profiles (Figure 1C and Figure S1A,B). Compared with sham controls, CLP lungs exhibited increased proportions of neutrophils and mesenchymal populations, along with alterations in endothelial and epithelial cell populations. Notably, mononuclear phagocytes (MPS)—particularly macrophages—were markedly enriched following CLP induction (Figure 1D,E). Notably, KAT6A expression in macrophages was significantly elevated in the CLP group (Figure 1F). Further stratification of macrophage subsets demonstrated that KAT6A was preferentially enriched in M1 macrophages (Figure 1G,H and Figure S1C,D).

To validate KAT6A protein expression in macrophages, we performed immunofluorescence staining on purified bone marrow–derived macrophages (BMDMs, Figure S1E). The staining confirmed that KAT6A is expressed in macrophages and displays a predominant nuclear staining pattern (Figure 1I). Both Western blotting and quantitative real-time PCR (qPCR) confirmed that KAT6A expression was significantly elevated in lung tissues from 12 h CLP-induced sepsis mice compared to sham-operated mice (Figure 1J,K). Together, these data indicate that KAT6A is upregulated during sepsis and enriched in inflammatory macrophages, suggesting its relevance to macrophage responses in sepsis.

### 3.2. KAT6A Inhibition Suppresses Glycolysis and Reprograms Macrophage Metabolic Activity

**Figure 2:**
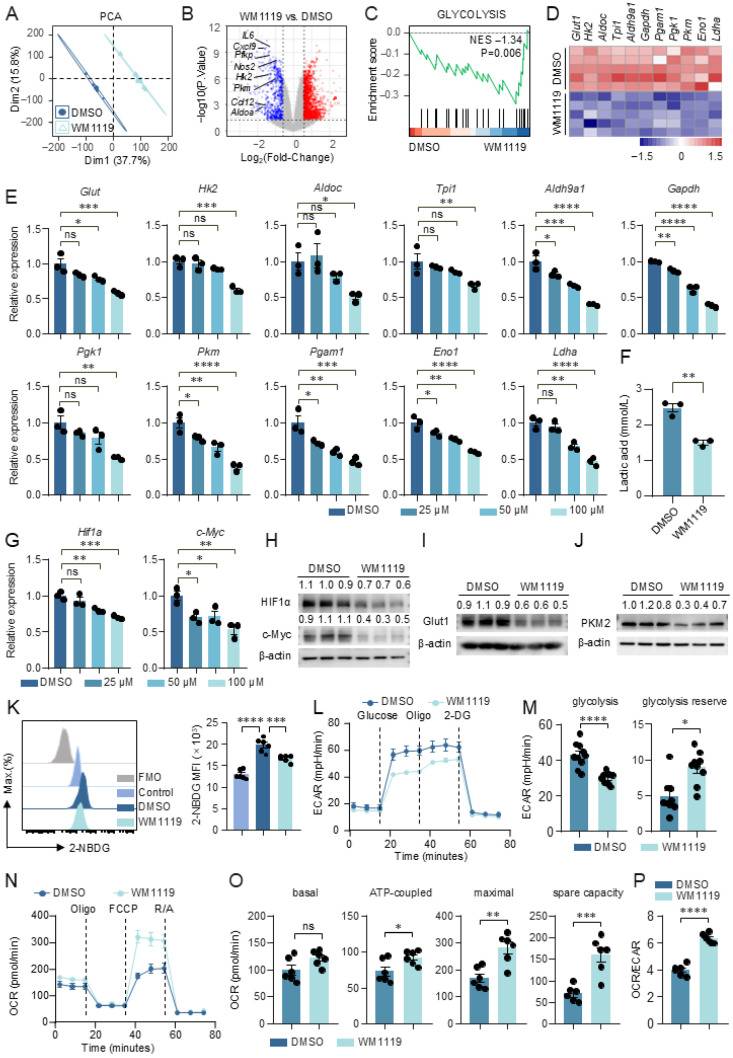
KAT6A inhibition suppresses glycolysis and reprograms macrophage metabolic activity. (**A**–**F**) PMs from C57BL/6J mice were pretreated with WM1119 or DMSO for 1 h, followed by stimulation with lipopolysaccharide (LPS, 100 ng/mL) for 6 h. Bulk RNA sequencing was performed on PMs to identify differentially expressed genes (DEGs) and enriched gene sets (n = 5). (**A**) Principal component analysis (PCA) of samples in different groups. (**B**) Volcano plot for DEGs. (**C**) Gene Set Enrichment Analysis (GSEA) plot showing enrichment score and normalized enrichment scores (NES) for glycolysis. (**D**) Heatmap shows expression of glycolytic genes. (**E**) Gene expression of glucose transporter 1 (*Glut1*), hexokinase (*Hk*) *2*, *Aldoc*, *Tpi1*, *Aldh9a1*, *Gapdh*, *Pgk1*, *Pkm*, *Pgam1*, *Eno1* and *Ldha* was measured by qPCR in PMs. Data are presented as fold change relative to the DMSO group (n = 3). (**F**) Lactate concentration in culture supernatants (n = 3). (**G**) Gene expression of *Hif1α* and *c-Myc* was measured by qPCR (n = 3). (**H**–**J**) Bone marrow-derived macrophages (BMDMs) from C57BL/6J mice were pretreated with WM1119 (100 μM) or DMSO for 1 h, followed by stimulation with LPS (100 ng/mL) for 24 h. (**H**) HIF1α and c-Myc expression were measured by Western blot, quantification of protein expression levels normalized to β-actin (n = 3). (**I**,**J**) GLUT1 and pyruvate kinase M2 (PKM2) expression was measured by Western blot, quantification of protein expression levels normalized to β-actin (n = 3). (**K**–**P**) BMDMs from C57BL/6J mice were pretreated with WM1119 (100 μM) or DMSO for 1 h, followed by stimulation with LPS (100 ng/mL) for 6 h. (**K**) BMDMs were incubated with 2-NBDG, a fluorescent glucose analogue, and glucose uptake was measured by flow cytometry. Mean fluorescence intensity (MFI) is shown (n = 6). (**L**–**P**) Extracellular acidification rate (ECAR) and oxygen consumption rate (OCR) were assessed with a Seahorse XF96 analyzer. (**L**,**M**) ECAR was measured following sequential injections of glucose, oligomycin (Oligo), and 2-deoxyglucose (2-DG), and the derived parameters for glycolysis and glycolytic capacity were quantified (n = 10). (**N**,**O**) OCR was determined after consecutive addition of Oligo, carbonyl cyanide-p-trifluoromethoxyphenylhydrazone (FCCP), and rotenone/antimycin A (R/A), with analyses summarizing basal respiration, ATP-linked respiration, maximal respiration, and spare respiratory capacity (n = 6). (**P**) Ratio of OCR to ECAR. Data are mean ± SEM. * *p* < 0.05, ** *p* < 0.01, *** *p* < 0.001, **** *p* < 0.0001. Statistics were done by one-way ANOVA followed by adjustments for multiple comparisons in panels (**E**,**G**,**K**), and two-tailed unpaired Student’s *t* test in panels (**F**,**M**,**O**,**P**). ns, not significant. Original Western blot images can be found in Figure S9.

Glycolysis is a key metabolic pathway that supports macrophage activation [37,38]. To determine whether KAT6A inhibition affects this metabolic program, we performed bulk RNA-seq on LPS-stimulated peritoneal macrophages (PMs) treated with WM1119. Flow cytometric analysis confirmed the high purity of the isolated PMs (Figure S2A). Principal component analysis (PCA) of the transcriptomes revealed a clear separation between WM1119- and DMSO-treated cells, with the first two dimensions (Dim1: 37.7%; Dim2: 15.8%) accounting for 53.5% of the total variance (Figure 2A). Differential expression analysis showed that WM1119 downregulated multiple glycolytic genes (*Pfkp*, hexokinase (*Hk*) *2*, *Pkm*, *Aldoa*) as well as pro-inflammatory and chemokine genes (*Il-6*, *Nos2*, *Cxcl9*, *Ccl12*) (Figure 2B). Gene set enrichment analysis (GSEA) further showed that the glycolysis pathway and several inflammation-related pathways, including interferon gamma response and IL-6-JAK-STAT3 signaling, were downregulated in WM1119-treated PMs (Figure 2C and Figure S2B,C). RNA-seq analysis showed that glycolytic gene expression was reduced in WM1119-treated PMs (Figure 2D). This decrease was further verified by qPCR, which demonstrated a dose-dependent inhibitory effect of WM1119 (Figure 2E). Similar results were obtained in BMDMs (Figure S3A–D). Consistent with the downregulation of glycolytic genes, WM1119 significantly attenuated lactate accumulation in LPS-stimulated macrophages (Figure 2F). The transcripts of hypoxia-inducible factor 1-alpha (HIF1α) and c-Myc, two key transcription factors in the metabolic pathway [37,39,40], were also downregulated by WM1119 (Figure 2G,H and Figure S3E). Glucose transporter 1 (GLUT1), the major glucose transporter upregulated during macrophage activation [41], and pyruvate kinase M2 (PKM2), a central regulator of glycolytic flux in inflammatory macrophages [37], were both markedly reduced at the protein level in WM1119-treated BMDMs (Figure 2I,J). In parallel, glucose uptake by BMDMs was significantly decreased by WM1119 (Figure 2K). These data indicate that KAT6A inhibition could lead to a reduction in glucose metabolism in activated macrophages. Notably, WM1119 did not induce cell death (Figure S4A,B).

After entering the cell, glucose is converted to pyruvate and then converted to lactate in the cytoplasm, or carbon dioxide and water in the mitochondria, generating ATP to meet cellular energy demands [42,43]. To further link KAT6A functions to glucose metabolism, we measured extracellular acidification rate (ECAR) and oxygen consumption rate (OCR) using Seahorse extracellular flux analysis. The glycolysis stress test results revealed that ECAR was decreased when BMDMs were treated with WM1119. Glycolysis and glycolytic capacity were both downregulated by WM1119 in BMDMs (Figure 2L,M). In contrast, Mito stress test analyses showed that ATP-linked respiration, maximal respiration, and spare respiratory capacity were significantly elevated upon KAT6A inhibition, while basal respiration exhibited an upward trend (Figure 2N,O). The OCR/ECAR ratio also significantly increased (Figure 2P). LPS stimulation-induced mitochondrial elongation was not altered by KAT6A inhibition (Figure S5A,B). Together, these findings demonstrate that KAT6A inhibition reprograms macrophage metabolism by suppressing glycolysis and enhancing mitochondrial respiration, independently of changes in mitochondrial morphology.

### 3.3. KAT6A Inhibition Modulates Macrophage Inflammatory Cytokine Production and M1 Polarization

**Figure 3:**
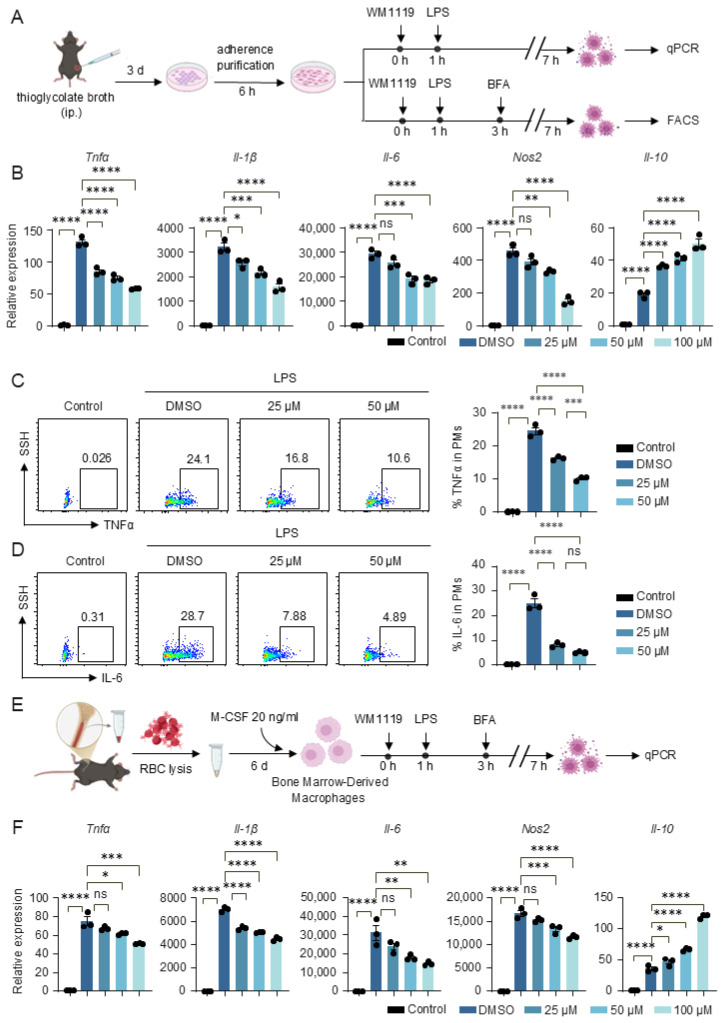
KAT6A inhibition attenuates inflammatory response in macrophages. (**A**–**D**) PMs and (**E**,**F**) BMDMs from C57BL/6J mice were pretreated with WM1119 at the indicated concentrations or DMSO for 1 h, followed by stimulation with LPS (100 ng/mL) for 6 h. (**A**) Experimental scheme in PMs. (**B**) qPCR analysis of *Tnfα*, *Il-1β*, *Il-6*, *Nos2*, *Il-10* (n = 3). (**C**,**D**) Flow cytometric analysis of TNFα and IL-6 production in F4/80^+^CD11b^+^ cells (n = 3). (**E**) Experimental scheme in BMDMs. (**F**) qPCR analysis of *Tnfα*, *Il-1β*, *Il-6*, *Nos2*, *Il-10* (n = 3). Data are mean ± SEM. * *p* < 0.05, ** *p* < 0.01, *** *p* < 0.001, **** *p* < 0.0001. Statistics were done by one-way ANOVA followed by adjustments for multiple comparisons in panels (**B**–**D**,**F**). ns, not significant.

To determine whether KAT6A-dependent metabolic regulation influences macrophage effector functions, we first assessed inflammatory cytokine expression in PMs. Consistent with the metabolic suppression observed upon KAT6A inhibition, WM1119 markedly reduced the mRNA levels of the pro-inflammatory genes *Tnfα*, *Il-1β*, *Il-6* and *Nos2* while increasing the anti-inflammatory cytokine *Il-10* in PMs (Figure 3A,B). Flow cytometry further confirmed decreased TNFα and IL-6 protein production in PMs following WM1119 treatment (Figure 3C,D). We next evaluated BMDMs and observed a similar pattern, with WM1119 decreasing *Tnfα*, *Il-1β*, *Il-6* and *Nos2* expression and elevating *Il-10* (Figure 3E,F), indicating that the metabolic shift induced by KAT6A inhibition consistently dampens inflammatory macrophage activation.

**Figure 4:**
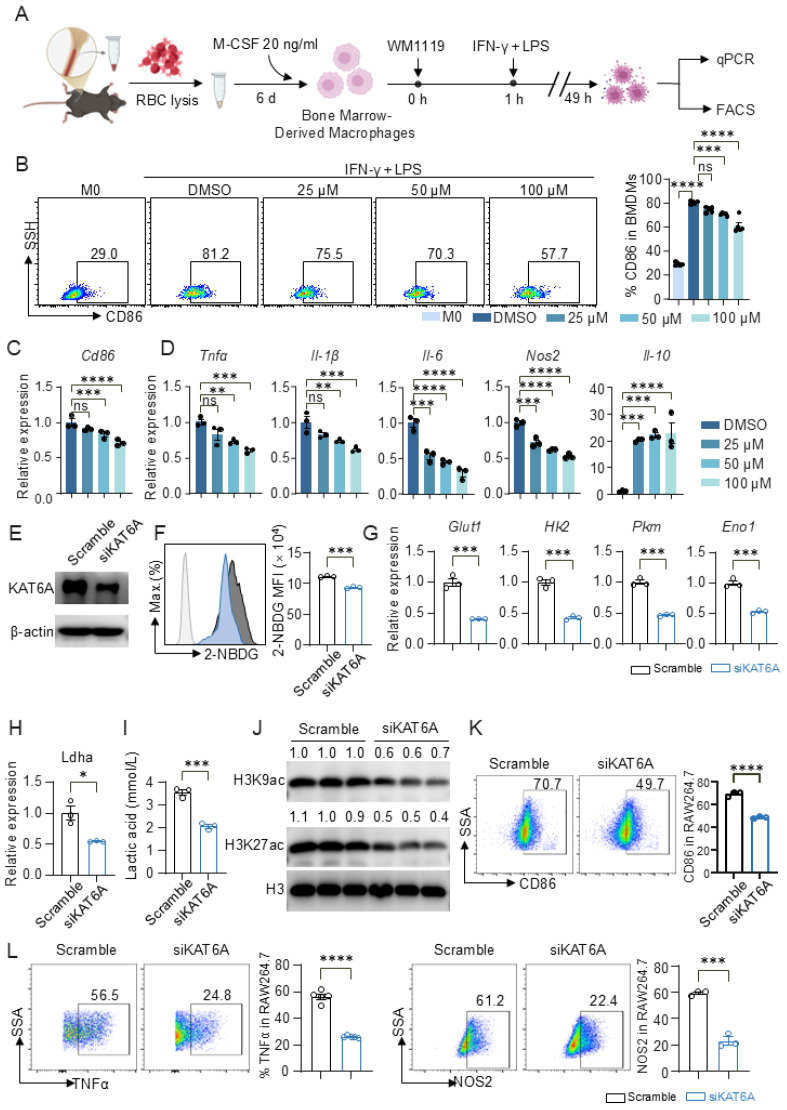
KAT6A inhibition suppresses M1 macrophage polarization. BMDMs were pretreated with WM1119 at the indicated concentrations or DMSO for 1 h, followed by stimulation with LPS (100 ng/mL) and interferon-gamma (IFN-γ) (50 ng/mL) for 48 h. (**A**) Experimental scheme. (**B**) Representative flow cytometric analysis of surface markers CD86 expression on F4/80^+^ BMDMs (n = 5). (**C**,**D**) qPCR analysis of *Cd86*, *Tnfα*, *Il-1β*, *Il-6*, *Nos2*, *Il-10* expression (n = 3). RAW264.7 macrophages transfected with siKAT6A or scramble control were stimulated with LPS and IFN-γ for 48 h. (**E**) Knockdown of KAT6A was confirmed by Western blot (n = 3). (**F**) Representative flow cytometric analysis and quantification of 2-NBDG uptake (n = 3). (**G**,**H**) qPCR analysis of glycolytic genes *Glut1*, *Hk2*, *Pkm*, *Eno1* and *Ldha* (n = 3). (**I**) Lactate concentration in culture supernatants (n = 3). (**J**) Immunoblot analysis of H3K9ac and H3K27ac acetylation. Representative bands of six biologically independent replicates. (**K**) Representative flow cytometric analysis and quantification of CD86 expression in RAW264.7 macrophages (n = 3). (**L**) Representative flow cytometric analysis and quantification of intracellular TNFα and NOS2 expression (n = 3). All data are mean ± SEM. * *p* < 0.05, ** *p* < 0.01, *** *p* < 0.001, **** *p* < 0.0001. Statistics were done by one-way ANOVA followed by adjustments for multiple comparisons in panels (**B**–**D**) and two-tailed unpaired Student’s *t* test in panels (**F**–**I**,**K**,**L**). ns, not significant. Original Western blot images can be found in Figure S9.

Given that KAT6A inhibition reduced glycolysis and inflammatory cytokine production, we next assessed its impact on macrophage polarization. LPS and the proinflammatory cytokine IFN-γ induce the differentiation of M1 macrophages, a process commonly known as classical activation [44]. Upon M1 polarization induced by IFN-γ and LPS, WM1119 markedly reduced the proportion of CD86^+^ macrophages in a dose-dependent manner (Figure 4A,B). At the transcriptional level, *Cd86* expression was similarly decreased by WM1119 (Figure 4C). WM1119 also suppressed the induction of M1-associated pro-inflammatory genes, including *Tnfα*, *Il-1β*, *Il-6* and *Nos2*, while increasing the anti-inflammatory cytokine *Il-10* (Figure 4D). We observed similar effects in PMs, where WM1119 consistently reduced M1-associated gene expression and increased *Il-10* upon IFN-γ and LPS stimulation (Figure S6A–C), indicating that the regulatory role of KAT6A in M1 polarization is conserved across macrophage types.

To genetically validate these findings, we knocked down KAT6A via siRNA in RAW264.7 macrophages (Figure 4E and Figure S6D) and induced M1 polarization with IFN-γ and LPS. siKAT6A significantly reduced glucose uptake as measured by 2-NBDG incorporation (Figure 4F). At the transcriptional level, the expression of glycolytic genes (*Glut1*, *Hk2*, *Pkm*, *Eno1* and *Ldha*) was markedly downregulated (Figure 4G–H), and extracellular lactate levels were correspondingly reduced (Figure 4I). Mechanistically, KAT6A knockdown decreased global H3K9ac and H3K27ac levels without affecting total H3 (Figure 4J), suggesting that KAT6A supports pro-inflammatory gene expression through histone acetylation. Accordingly, siKAT6A significantly decreased CD86^+^ cell proportions (Figure 4K) and reduced the frequencies of TNFα^+^ and NOS2^+^ cells (Figure 4L), further supporting that KAT6A promotes M1 macrophage polarization.

### 3.4. KAT6A Inhibition Suppresses PI3K-AKT-mTOR Signaling and M1 Macrophage Polarization

**Figure 5:**
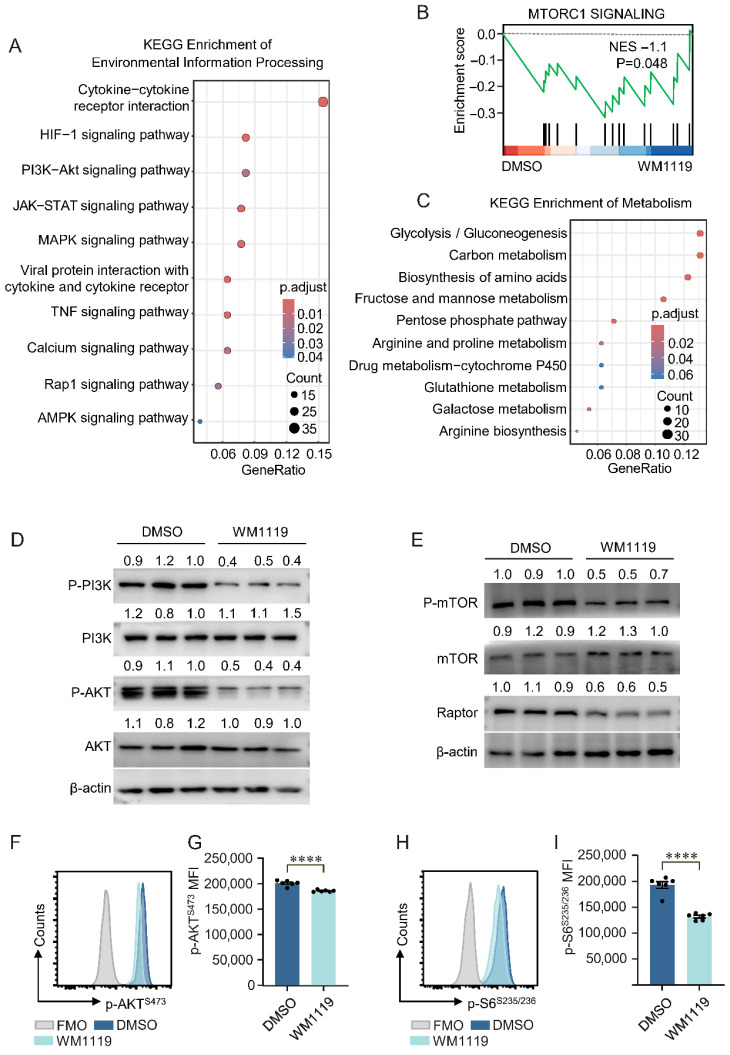
KAT6A inhibition is associated with reduced PI3K-AKT-mTOR signaling and M1 macrophage polarization. (**A**) KEGG enrichment of environmental information processing in down-regulated DEGs derived from WM1119-treated PMs. DEGs were defined as described in Figure 2. (**B**) GSEA plot showing enrichment score and NES for mTOR complex 1 (mTORC1) signaling. (**C**) KEGG enrichment of metabolism in down-regulated genes. (**D**–**I**) BMDMs from C57BL/6J mice were pretreated with WM1119 (100 μM) or DMSO for 1 h, followed by stimulation with LPS (100 ng/mL) for 24 h. Western blot images of the protein expression of (**D**) p-PI3K^p85^, PI3K, p-AKT^S473^ and AKT and (**E**) p-mTOR^S2448^, mTOR, and Raptor in the indicated groups. Quantification of protein expression levels normalized to β-actin (n = 3). BMDMs were stained for p-AKT^S473^ (**F**,**G**) and p-S6^S235/236^ (**H**,**I**) and analyzed by flow cytometry. (n = 6). All data are mean ± SEM. **** *p* < 0.0001. Statistics were done by a two-tailed unpaired Student’s *t* test in panels (**G**,**I**). Original Western blot images can be found in Figure S9.

As PI3K-AKT-mTOR signaling is a key pathway supporting macrophage activation and M1 polarization [45,46], we investigated whether this axis is affected by KAT6A inhibition. Kyoto Encyclopedia of Genes and Genomes (KEGG) pathway analysis showed that genes downregulated by WM1119 were significantly enriched in pathways involved in macrophage activation and polarization, including HIF-1, PI3K-AKT, JAK-STAT and MAPK signaling (Figure 5A). GSEA further demonstrated downregulation of the mTOR complex 1 (mTORC1) signaling pathway in WM1119-treated macrophages (Figure 5B). KEGG analysis also indicated enrichment of metabolic pathways broadly associated with cellular energy utilization (Figure 5C).

To validate these transcriptomic observations, we examined the activity of the PI3K-AKT-mTORC1 axis at the protein level. Western blot analysis showed that WM1119 markedly reduced phosphorylated PI3K^p85^ and phosphorylated AKT^S473^, without altering total protein levels (Figure 5D). The phosphorylation of mTOR as well as the expression of the mTORC1-associated subunit Raptor were similarly decreased following KAT6A inhibition (Figure 5E). Flow cytometric analysis further confirmed reduced PI3K-AKT-mTORC1 signaling, as reflected by decreased p-AKT^S473^ and p-S6^S235/236^ in WM1119-treated BMDMs (Figure 5F–I). Together, these findings indicate that KAT6A inhibition is associated with reduced PI3K-AKT-mTORC1 activity, which may contribute to the suppression of macrophage activation and M1 polarization.

### 3.5. KAT6A Inhibition Protects Against Organ Injury at 12 h After CLP-Induced Sepsis

**Figure 6:**
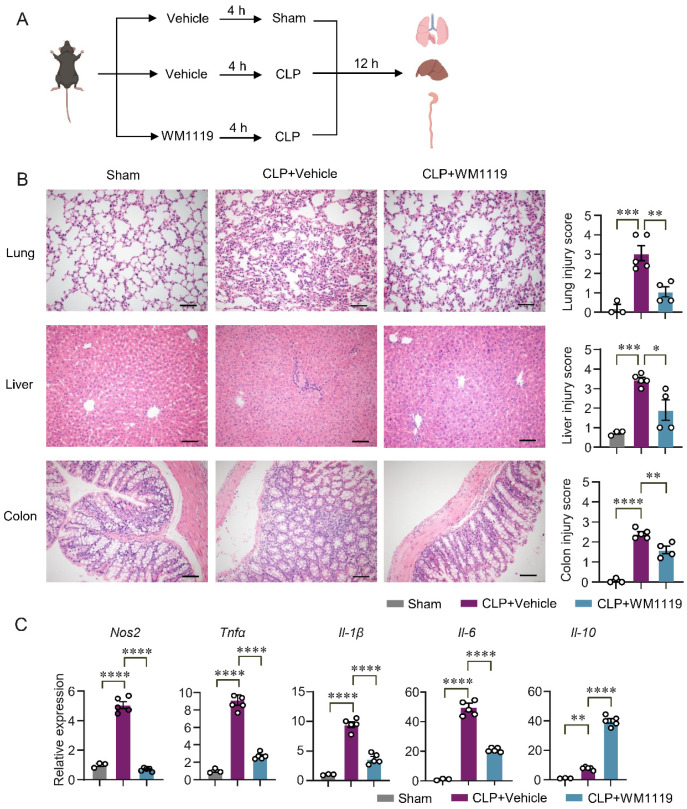
KAT6A inhibition protects against organ injury at 12 h after CLP-induced sepsis. (**A**) Scheme of the mouse experiment. C57BL/6J mice were randomly assigned to three groups. 4 h after receiving vehicle or WM1119 (50 mg/kg), mice were subjected to sham or CLP surgery. All mice were euthanized 12 h after surgery for organ injury assessment. (**B**) H&E staining and histological score of lung, liver and colon sections from the indicated groups. Scale bar: 100 µm (lung, liver), Scale bar: 50 µm (colon) (n = 3 for sham group, n = 5 for CLP group, n = 4 for CLP+WM1119 group). (**C**) qPCR of cytokine genes (*Tnfα*, *Il-1β*, *Il-6*, *Nos2*, *Il-10*) in lung tissues from the indicated groups (n = 3 for sham group, n = 5 for CLP and CLP+WM1119 group). All data are mean ± SEM. * *p* < 0.05, ** *p* < 0.01, *** *p* < 0.001, **** *p* < 0.0001. Statistics were done by one-way ANOVA followed by adjustments for multiple comparisons in panels (**B**,**C**).

To evaluate the role of KAT6A in sepsis pathogenesis in vivo, we established a polymicrobial sepsis model induced by cecal ligation and puncture (CLP). C57BL/6J mice were pretreated with WM1119 (50 mg/kg) [47] or vehicle for 4 h before CLP or sham surgery, and tissues were collected 12 h after CLP (Figure 6A). Histopathological examination of H&E-stained sections revealed severe lung, liver and colon injury in CLP mice, whereas WM1119 treatment markedly alleviated lung damage, reduced hepatic necrosis, and attenuated colonic injury, as reflected by significantly lower organ injury scores (Figure 6B), indicating a protective effect of KAT6A inhibition against early septic injury. Consistent with these histologic improvements, qPCR analysis of lung tissues showed that WM1119 significantly downregulated the CLP-induced expression of pro-inflammatory genes (*Tnfα*, *Il-1β*, *Il-6* and *Nos2*) while upregulating the anti-inflammatory cytokine *Il-10* (Figure 6C), supporting an immunomodulatory role of KAT6A in early sepsis. Notably, KAT6A inhibition did not markedly alter the overall composition of innate and adaptive immune cell subsets—including T cells, B cells, eosinophils, monocytes, neutrophils and dendritic cells—in lungs from septic mice (Figure S7A,B), suggesting that its protective effects are mediated primarily through functional reprogramming rather than depletion of immune cells.

### 3.6. KAT6A Inhibition Protects Mice from Organ Injury and Promotes Bacterial Clearance at 24 h After CLP-Induced Sepsis

**Figure 7:**
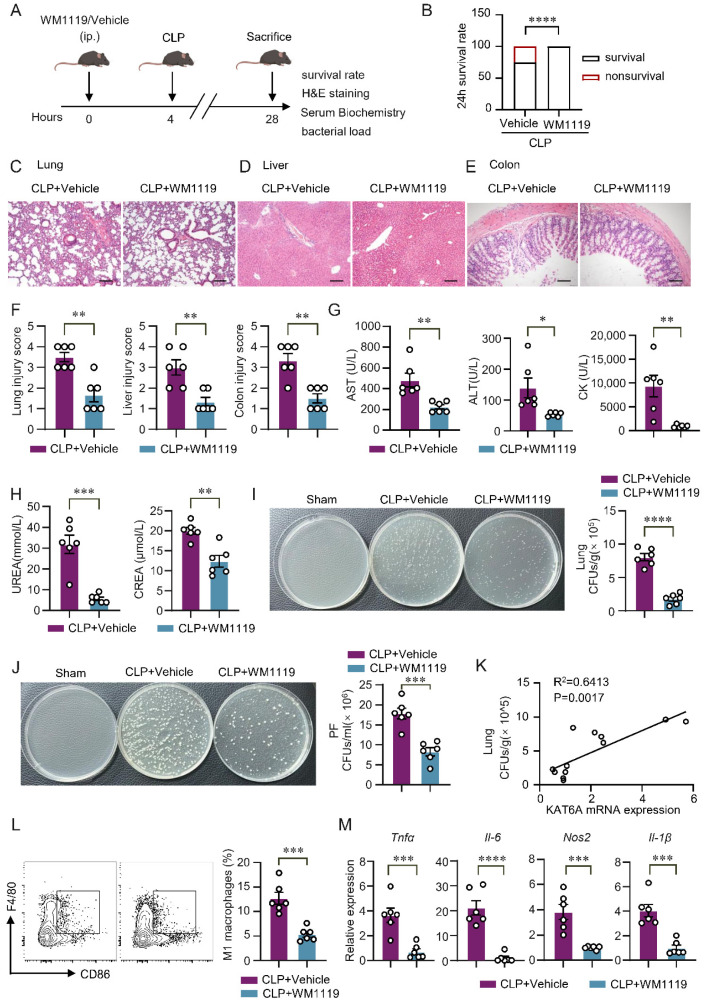
KAT6A inhibition protects against organ injury and promotes bacterial clearance at 24 h after CLP-induced sepsis. (**A**) Scheme of the mouse experiment. Mice were pretreated with WM1119 (50 mg/kg) or vehicle 4 h before CLP. Mice were euthanized 24 h after CLP, and tissues were harvested for analysis. (**B**) 24 h survival rate was recorded. (**C**–**E**) Representative H&E staining images of lung, liver and colon from septic mice treated with WM1119 or vehicle, with their respective histological scores quantified in (**F**) (n = 6). Scale bar: 100 μm (**C**,**D**), Scale bar: 50 μm (**E**). (**G**,**H**) Serum concentrations of aspartate aminotransferase (AST), alanine aminotransferase (ALT), creatine kinase (CK), urea, and creatinine (Crea) were measured in the indicated groups (n = 6). Bacterial burden was quantified as colony-forming units (CFU) per mL for peritoneal fluid (**I**) (n = 6) and CFU per gram for lung tissues (**J**) (n = 6). (**K**) Correlations between KAT6A mRNA expression levels and lung CFUs (n = 6 for each group). (**L**) The percentage of M1 macrophages (F4/80^+^CD86^+^) in septic lung tissues treated with WM1119 or vehicle (n = 6). (**M**) qPCR of cytokine genes (*Tnfα*, *Il-6*, *Nos2*, *Il-1β*) in lung tissues treated with WM1119 or vehicle (n = 6). All data are mean ± SEM. * *p* < 0.05, ** *p* < 0.01, *** *p* < 0.001, **** *p* < 0.0001. Statistics were done by a two-tailed unpaired Student’s *t* test in panels (**B**,**F**,**G**–**J**,**L**,**M**). Correlation was assessed by the Pearson correlation coefficient in panel (**K**).

To assess whether KAT6A inhibition provides sustained protection during later-stage sepsis, we extended the CLP model to 24 h. Mice were pretreated with WM1119 (50 mg/kg) or vehicle 4 h before CLP (Figure 7A). KAT6A inhibition significantly improved 24 h survival compared with vehicle-treated septic mice (Figure 7B). Histopathological analysis revealed pronounced lung, liver, and colon injury in vehicle-treated mice, whereas WM1119 markedly attenuated tissue damage (Figure 7C–F), consistent with the more severe pathology typically observed at 24 h compared with the early phase of sepsis. We next evaluated systemic organ dysfunction. Serum aspartate aminotransferase (AST), alanine aminotransferase (ALT), creatine kinase (CK), urea, and creatinine (Crea) levels were all significantly reduced by WM1119 treatment (Figure 7G,H), indicating broad mitigation of CLP-induced hepatic, myocardial and renal injury. Because improved organ integrity in sepsis is often associated with better bacterial control, we quantified microbial burdens in peritoneal fluid and lung tissues. WM1119 significantly reduced bacterial load in both compartments (Figure 7I,J). Moreover, lung bacterial counts positively correlated with KAT6A mRNA expression (Figure 7K), suggesting that elevated KAT6A is linked to impaired host antibacterial defense.

Because our in vitro experiments demonstrated that KAT6A promotes M1 macrophage polarization (Figure 4), we next examined whether this regulatory role is preserved in vivo using a 24 h CLP model. Flow cytometry analysis revealed a marked reduction in CD86^+^ M1 macrophages in WM1119-treated mice (Figure 7L, see gating strategy in Figure S8), indicating suppressed M1 polarization. Consistently, expression of M1-associated genes (*Tnfα*, *Il-1β*, *Il-6*, and *Nos2*) was reduced, whereas *Il-10* expression increased (Figure 7M). Together, these findings identify KAT6A as a regulator of M1 macrophage polarization in vivo and reveal that its inhibition provides significant protection in sepsis.

## 4. Discussion

Macrophages are central regulators of host defense and inflammatory injury in sepsis-induced ALI [48,49,50,51]. Although macrophage-driven inflammation is a hallmark of sepsis [52], the upstream molecular mechanisms that govern their activation remain incompletely understood. Although KAT6A has recently been implicated in regulating macrophage inflammatory responses in non-pulmonary diseases such as periodontal inflammation [24], its role in systemic inflammation and sepsis-induced ALI has not been explored. In this study, we identify KAT6A as a sepsis-induced epigenetic regulator of macrophage activation. Re-analysis of published transcriptomic datasets showed that KAT6A expression is increased in peripheral blood samples from patients with sepsis and in M1 macrophage subsets from septic mice, and our experimental data further demonstrated upregulated KAT6A in lung tissues from CLP-treated mice. Pharmacological inhibition of KAT6A with the selective inhibitor WM1119 markedly attenuated lung inflammation, improved bacterial clearance, and prolonged survival in CLP-induced sepsis, establishing KAT6A as a previously unrecognized epigenetic mediator of macrophage-driven lung injury.

Classically activated M1 macrophages undergo a well-established shift toward aerobic glycolysis [53], which fuels the rapid production of pro-inflammatory mediators such as IL-1β, IL-6, TNFα and NOS2 [54]. Consistent with this, we found that KAT6A appears to be important for maintaining both glycolytic activity and inflammatory gene expression in macrophages. In LPS-stimulated BMDMs, inhibition of KAT6A reduced glycolytic flux and downregulated glycolysis-associated genes, accompanied by decreased expression of canonical M1 cytokines including *Il-1β*, *Il-6*, *Tnfα*, and *Nos2*. These findings suggest that KAT6A activity may contribute to sustaining the glycolysis-dependent inflammatory state of M1 macrophages. In vivo, WM1119 treatment suppressed pulmonary cytokine production and inflammatory macrophage activation in CLP-induced sepsis, underscoring the essential role of KAT6A in macrophage-driven lung inflammation. In our CLP model, we therefore assessed cytokine release at 12 h as an early inflammatory time point and polarization markers at 24 h, when innate immune tolerance has been reported to emerge [55,56]. These temporal patterns suggest that KAT6A contributes to both the early secretory response and the subsequent stabilization of inflammatory polarization.

The maintenance of glycolytic and inflammatory programs in macrophages relies on the sustained activity of transcriptional regulators such as HIF1α and c-Myc, which coordinate the expression of glycolytic enzymes and pro-inflammatory mediators [57,58]. In our study, KAT6A inhibition led to the reduced expression of both HIF1α and c-Myc, indicating that KAT6A promotes macrophage activation in part through these factors. KAT6A inhibition was also associated with markedly reduced PI3K-AKT-mTOR signaling, a central metabolic pathway that reinforces glycolytic flux and stabilizes the M1 phenotype. These observations are consistent with prior studies showing that MOZ/KAT6A supports MYC-dependent transcriptional programs in hematopoietic malignancies, in part through its role in establishing active histone acetylation marks such as H3K9ac [59,60]. Importantly, genetic depletion of KAT6A via siRNA in RAW264.7 macrophages reduced global levels of H3K9ac and H3K27ac, two histone acetylation marks catalyzed by KAT6A, without affecting total H3 abundance, providing direct epigenetic evidence in macrophages. These findings support a model in which KAT6A creates a permissive chromatin state that sustains HIF1α/c-Myc activity and PI3K-AKT-mTOR-dependent metabolic reprogramming, thereby reinforcing the pro-inflammatory phenotype of M1 macrophages.

To complement the pharmacological findings with WM1119, we employed a genetic loss-of-function approach using siRNA-mediated KAT6A knockdown in RAW264.7 macrophages. siKAT6A recapitulated the key effects of WM1119. It reduced glucose uptake, suppressed glycolytic gene expression and extracellular lactate accumulation, and attenuated M1 polarization as evidenced by decreased CD86 surface expression and reduced intracellular TNFα and NOS2 levels. The convergence of pharmacological and genetic evidence across multiple macrophage models, including BMDMs, peritoneal macrophages, and RAW264.7 cells, supports the notion that KAT6A plays an important role in the metabolic and inflammatory programs associated with M1 macrophage polarization. These complementary approaches also mitigate concerns about off-target effects of WM1119 and support the specificity of the observed phenotypes.

The co-occurrence of reduced M1 polarization and improved bacterial clearance following KAT6A inhibition may initially appear paradoxical, as M1 macrophages are classically associated with antimicrobial responses [44]. However, in the context of polymicrobial sepsis, excessive and uncontrolled M1 activation is recognized as pathological rather than protective. Hyperactivated macrophages divert oxidative and biosynthetic resources toward cytokine overproduction at the expense of phagocytic efficiency, thereby impairing rather than supporting bacterial elimination [61]. KAT6A inhibition may therefore restore a more functionally balanced macrophage state that supports effective phagocytosis, consistent with emerging evidence that modulating macrophage metabolic programs can decouple cytokine overproduction from phagocytic function in sepsis [62].

Several limitations of this study should be acknowledged. First, we did not define the direct chromatin targets of KAT6A; thus, its locus-specific regulation of glycolytic and inflammatory genes remains to be fully elucidated. Second, although the CLP model recapitulates major features of sepsis [25,63], differences in immune heterogeneity and disease dynamics between mice and humans warrant further evaluation of KAT6A function in clinical settings. Third, our data demonstrate that KAT6A inhibition is accompanied by reduced PI3K-AKT-mTOR activity; however, whether KAT6A directly regulates this pathway or acts through intermediary effectors remains to be determined in future studies. Fourth, direct locus-specific evidence linking KAT6A-dependent histone acetylation to glycolytic gene promoters remains to be established by future ChIP-based analyses. Despite these considerations, our findings demonstrate that KAT6A is associated with PI3K-AKT-mTOR-dependent metabolic reprogramming, characterized by enhanced glycolysis and reduced oxidative phosphorylation, which supports the M1 pro-inflammatory phenotype that drives sepsis-induced lung injury. Pharmacologic inhibition of KAT6A reversed this metabolic program, attenuated inflammatory activation, and ameliorated tissue damage. Collectively, this study provides mechanistic insight into how KAT6A modulates macrophage metabolism and inflammation. Unlike broad immunosuppressive strategies, targeting KAT6A may offer a more selective approach to rebalance macrophage-driven inflammation while maintaining host antibacterial defense.

## 5. Conclusions

Our study demonstrated that KAT6A is upregulated in macrophages from septic mice and LPS-stimulated macrophages in vitro. KAT6A is associated with sepsis pathogenesis, and its upregulation correlates with enhanced glycolytic activity and M1 polarization. Therefore, targeting KAT6A could effectively alleviate organ injury and improve outcomes in sepsis by reprogramming macrophage function.

## Data Availability

The data used and/or analyzed during the current study are available from the corresponding author upon reasonable request.

## Supplementary Materials

The following supporting information can be downloaded at: https://www.mdpi.com/article/10.3390/biom16040609/s1, Figure S1: Characterization of macrophages; Figure S2: Enriched pathways in PMs; Figure S3: KAT6A inhibition downregulated expression of key glycolysis enzymes in BMDMs; Figure S4: KAT6A inhibition did not influence cell viability of macrophages; Figure S5: KAT6A inhibition did not affect mitochondrial length or morphology in macrophages; Figure S6: KAT6A inhibition downregulated proinflammatory M1-associated genes; Figure S7: Effect of KAT6A inhibition on immune cell subset composition; Figure S8: Gating strategy for flow cytometry analysis of macrophages in lung tissues; Figure S9: Uncropped gels for Western blot in Figure 1, Figure 2 and Figure 5; Table S1: List of qPCR primer sequences.

## Abbreviations

The following abbreviations are used in this manuscript:

ALI	Acute lung injury
KAT6A	Lysine acetyltransferase 6A
CLP	Cecal ligation and puncture
SPF	Specific pathogen-free
HATs	Histone acetyltransferases
HDACs	Histone deacetylases
UMAP	Uniform Manifold Approximation and Projection
PCA	Principal component analysis
GSEA	Gene set enrichment analysis
NES	Normalized enrichment score
KEGG	Kyoto Encyclopedia of Genes and Genomes
MPS	Mononuclear phagocytes
BMDMs	Bone marrow-derived macrophages
PMs	Peritoneal macrophages
MFI	Mean fluorescence intensity
LPS	Lipopolysaccharide
IFN-γ	Interferon-gamma
qPCR	Quantitative real-time PCR
H&E	Hematoxylin and eosin
BSA	Bovine serum albumin
AST	Aspartate aminotransferase
ALT	Alanine aminotransferase
CK	Creatine kinase
Crea	Creatinine
ECAR	Extracellular acidification rate
OCR	Oxygen consumption rate
HIF1α	Hypoxia-inducible factor 1-alpha
PKM2	Pyruvate Kinase M2
HK2	Hexokinase 2
GLUT1	Glucose Transporter 1
mTORC1	mTOR complex 1
CFUs	Colony-forming units
