# Supplementary material for: Lysine Acetyltransferase 6A Drives M1 Macrophage Polarization Through Metabolic Reprogramming in Sepsis-Induced Acute Lung Injury
Source: Biomolecules. 2026 Apr 20;16(4):609. doi: 10.3390/biom16040609 (PMC13113684; doi:10.3390/biom16040609)
Supplement: Supplementary file 1 [file biomolecules-16-00609-s001.zip › biomolecules-4177052-supplementary.pdf]

Supplementary Information

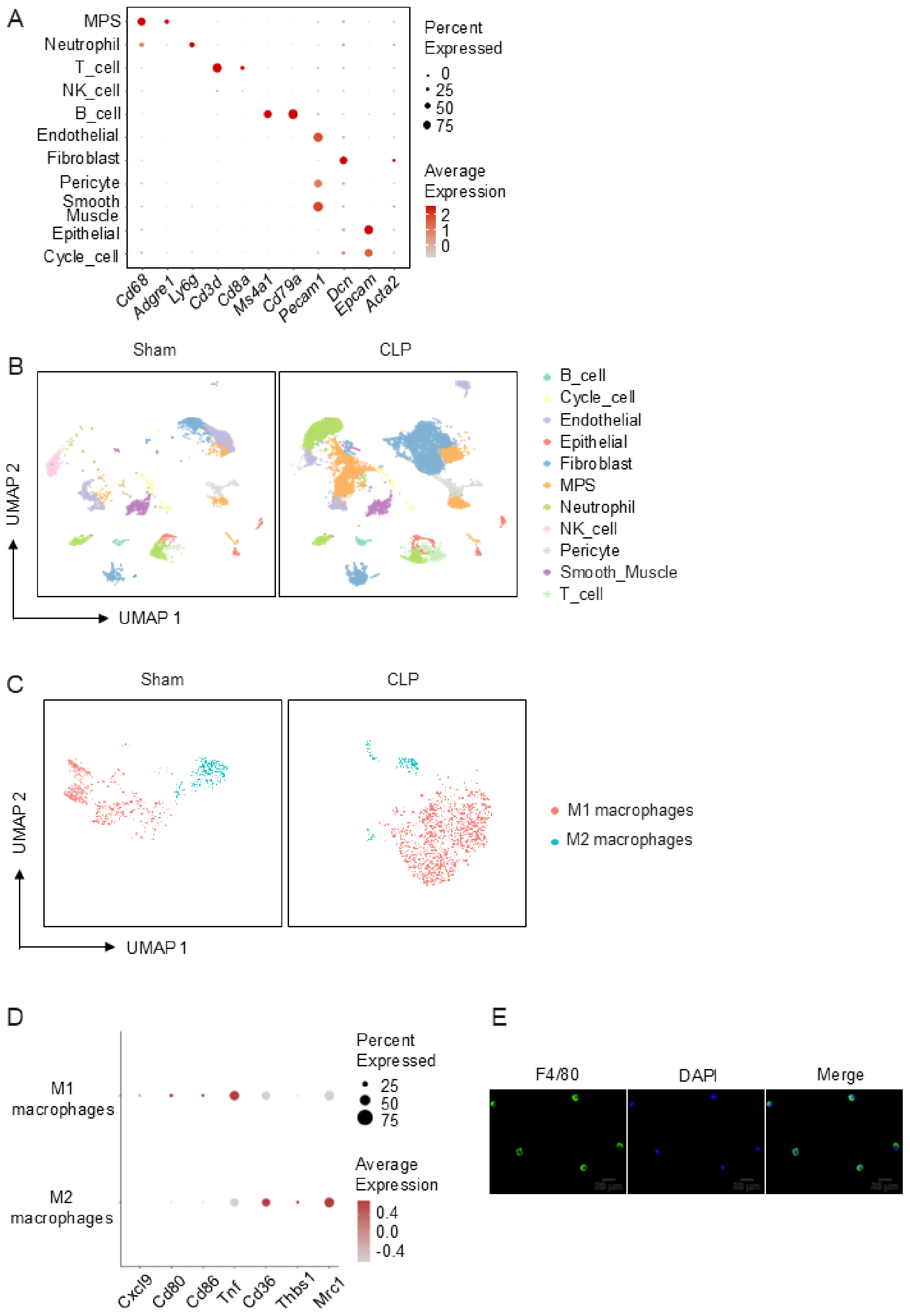

Figure S1: Characterization of lung cell populations and macrophages. (**A**) Dot plot of canonical marker gene expression across cell populations identified by

scRNA-seq of lung tissues from sham and CLP mice. (**B**) UMAP plots depicting cell type composition in sham and CLP lung tissues. (**C**) UMAP plots showing the distribution of M1 and M2 macrophage subpopulations in sham and CLP lung tissues. (**D**) Single-cell RNA-seq of lung tissues from sham and CLP mice identifying M1/M2 marker expression in macrophage subclusters. (**E**) Immunofluorescence staining of bone marrow-derived macrophages (BMDMs) demonstrated uniform positivity for F4/80 (green) and DAPI (blue). Scale bar: 20 μm.

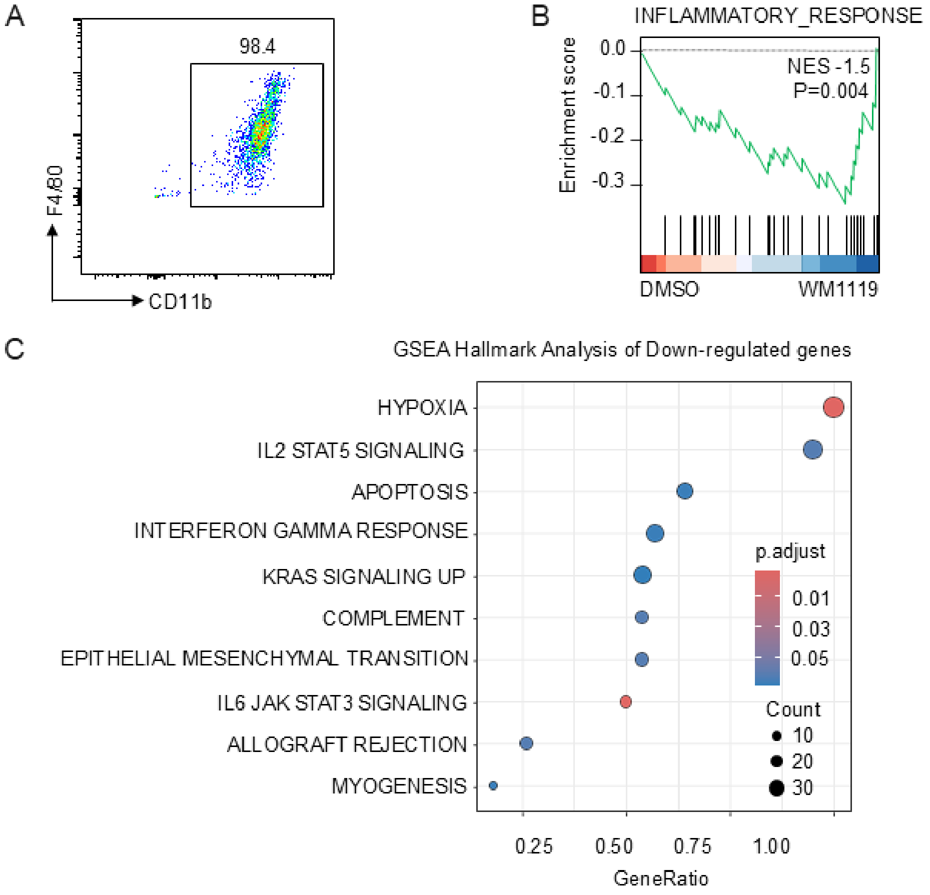

Figure S2: Enriched pathways in PMs. (**A**) Flow cytometry confirming F4/80$^+$CD11b$^+$ peritoneal macrophages (PMs). (**B**) Gene Set Enrichment

Analysis (GSEA) plot showing Enrichment Score and normalized enrichment scores (NES) for inflammatory response. (**C**) GSEA hallmark pathway enrichment of downregulated differentially expressed genes.

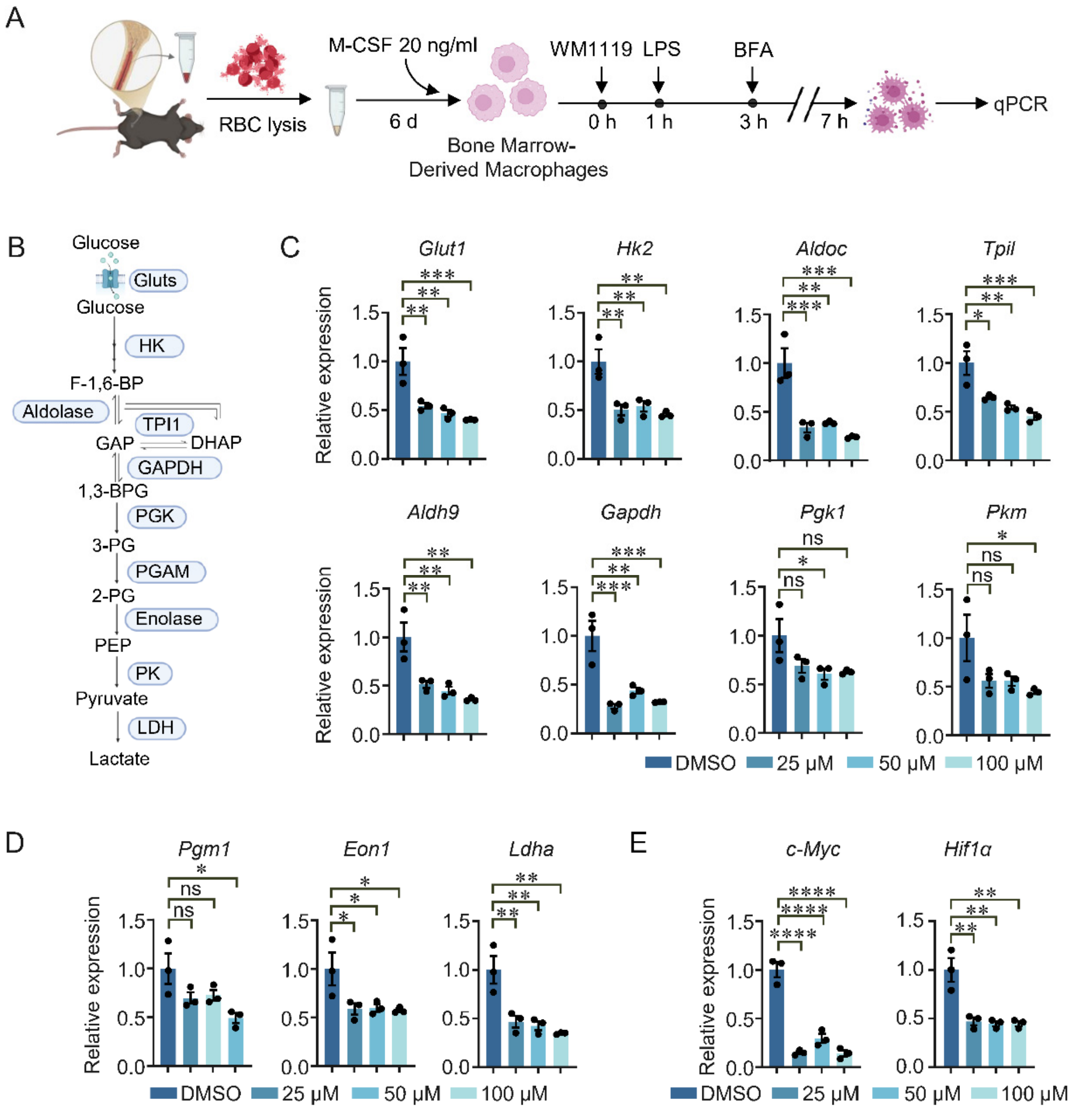

Figure S3: KAT6A inhibition downregulated expression of key glycolysis enzymes in BMDMs. (**A**) Experimental scheme. (**B**) A schematic illustration of a key enzyme in the glycolytic pathway. (Abbreviations: G-6-P: Glucose-6-phosphate; FBP: Fructose-1, 6-phosphate; GAP: Glyceraldehyde-3-phosphate;

1,3-BPG: 1,3-bisphosphoglycerate; 3-PG: 3-phosphoglycerate; 2-PG: 2-phosphoglycerate; PG: phosphoglycerate; DHAP: Dihydroxyacetone phosphate). (**C, D**). Gene expression of glucose transporter 1 (*Glut1*), hexokinase (*Hk*) 2, *Aldoc*, *Tpi1*, *Aldh9a1*, *Gapdh*, *Pgk1*, *Pkm*, *Pgam1*, *Eno1* and *Ldha* were measured by qPCR in BMDMs (n = 3). (**E**) Gene expression of *c-Myc*, *Hif1α* were measured by qPCR (n = 3). Data are mean ± SEM. *$p < 0.05$, **$p < 0.01$, ***$p < 0.001$, ****$p < 0.0001$. Statistics were done by one-way ANOVA with Tukey's post hoc test for multiple comparisons in panels C, D and E. ns, not significant.

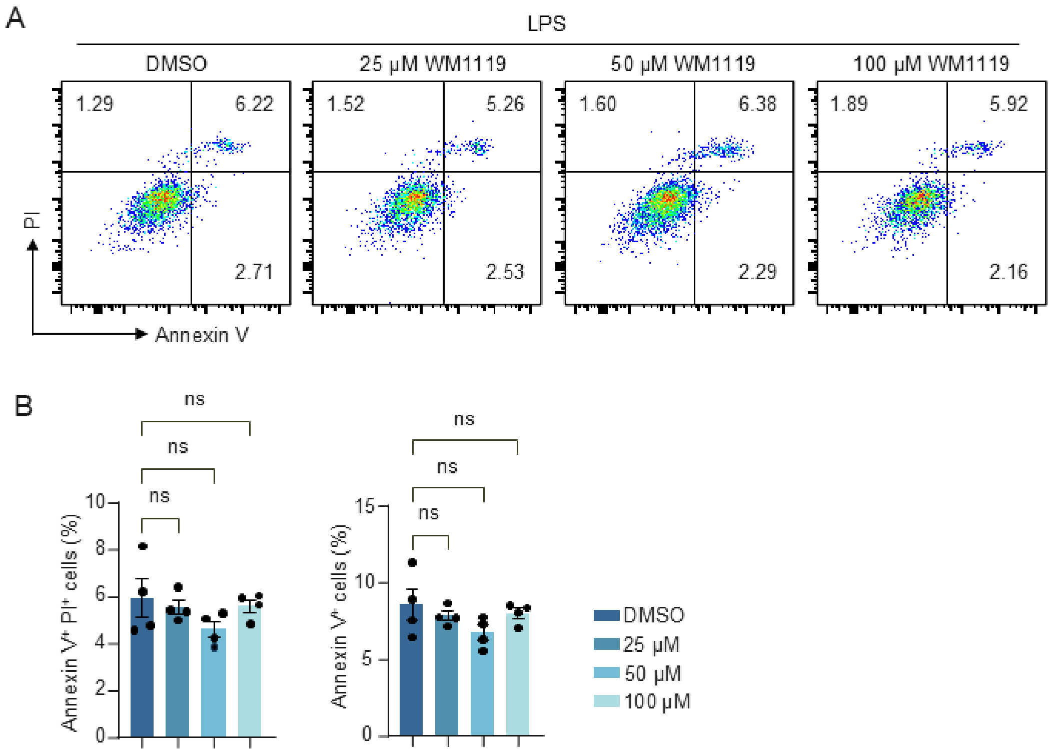

Figure S4: KAT6A inhibition did not influence cell viability of macrophages. (**A, B**) Viability of BMDMs was determined by Annexin-V/PI staining and analyzed

by flow cytometry (n=3). Data are mean ± SEM. Statistics were done by one-way ANOVA with Tukey's post hoc test for multiple comparisons in panel B. ns, not significant.

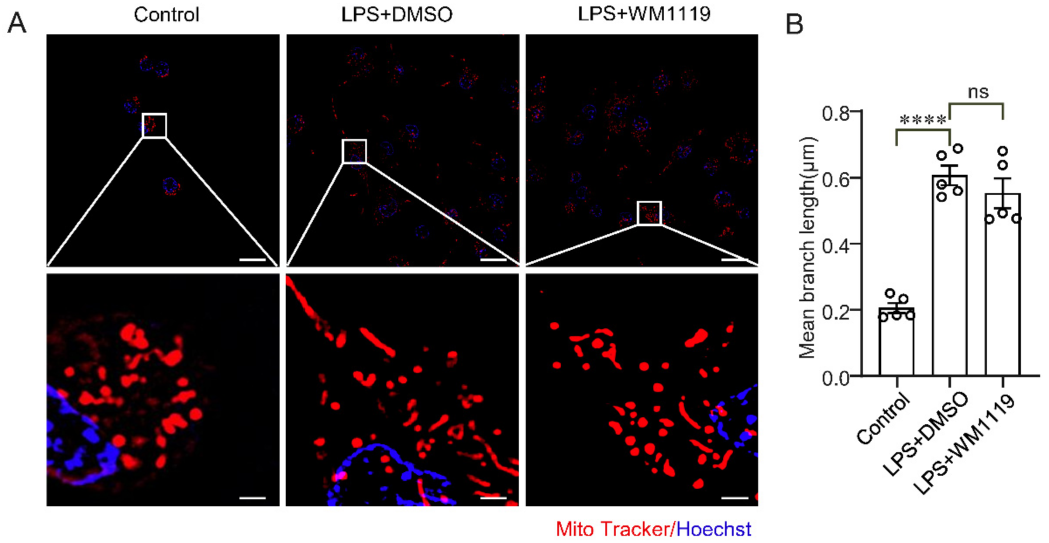

Figure S5: KAT6A inhibition did not affect mitochondrial length or morphology in macrophages. (**A**) BMDMs treated with WM1119 or DMSO and stimulated with LPS were stained with Hoechst (blue) and Mito Tracker Green (red). Representative images by confocal microscopy showed mitochondrial staining. scale bar: 10μm (top); scale bar, 2μm (bottom). (**B**) Mitochondrial branch length per cell was calculated and summarized from 5 independent samples, respectively. Data are mean ± SEM. ****p < 0.0001. Statistics were done by one-way ANOVA with Tukey's post hoc test for multiple comparisons in panel B. ns, not significant.

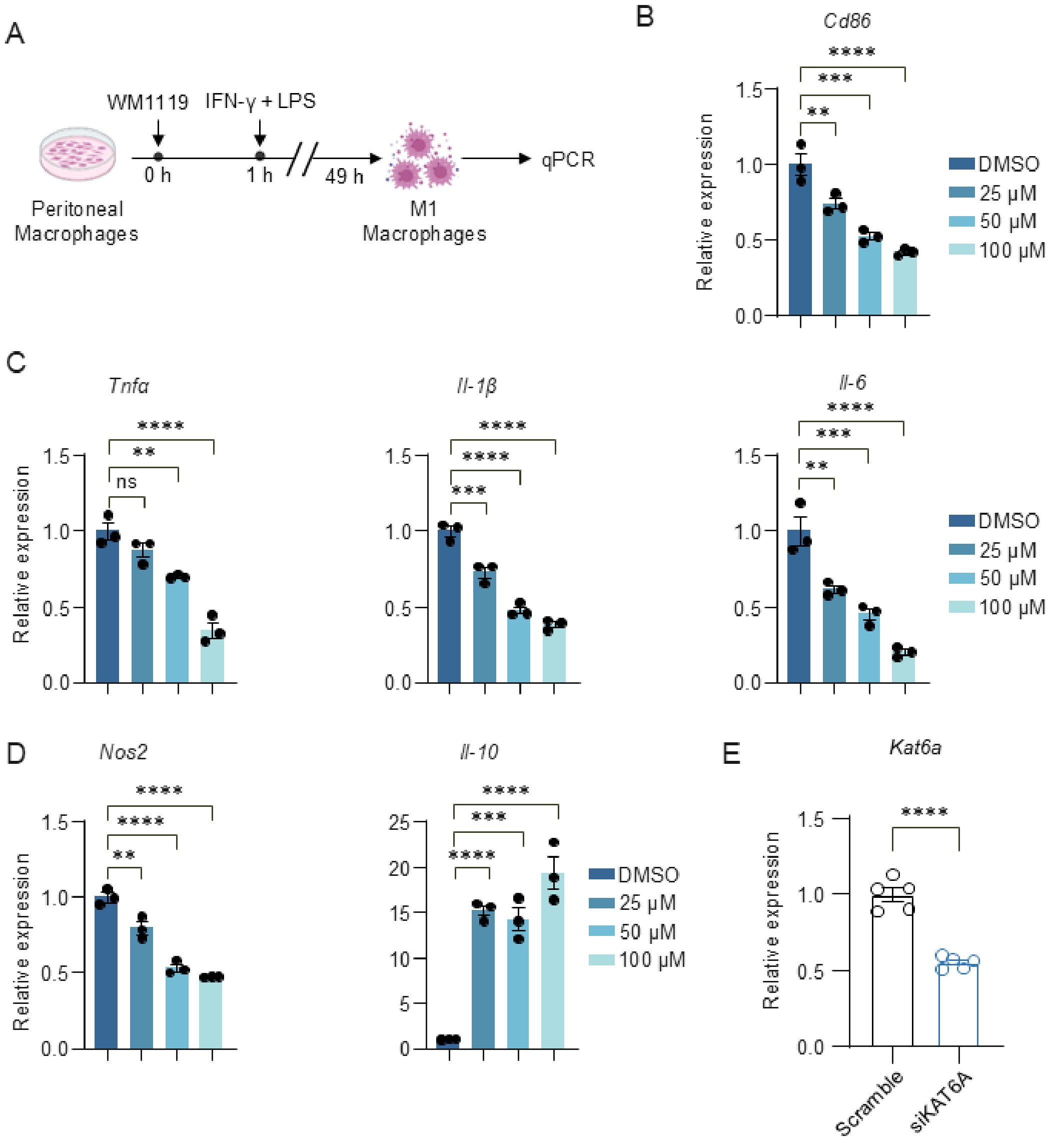

Figure S6: KAT6A inhibition downregulated proinflammatory M1-associated genes. PMs were pretreated with WM1119 at the indicated concentrations or DMSO for 1 h, followed by stimulation with IFN-γ (50ng/mL) and LPS (100ng/mL) for 48 h. (**A**) Experimental scheme. (**B, C, D**) qPCR analysis of *Cd86*, *Tnfα*, *Il-1β*, *Il-6*, *Nos2*, *Il-10* expression in PMs (n = 3). (**E**) qPCR analysis of Kat6a expression in LPS-stimulated RAW264.7 macrophages transfected with siKAT6A or scramble control (n = 5). Data are mean ± SEM. ***p* < 0.01, ****p* < 0.001, *****p* < 0.0001. Statistics were done by one-way ANOVA with

Tukey's post hoc test for multiple comparisons in panels B, C, D and two-tailed unpaired Student's t test in panel E. ns, not significant.

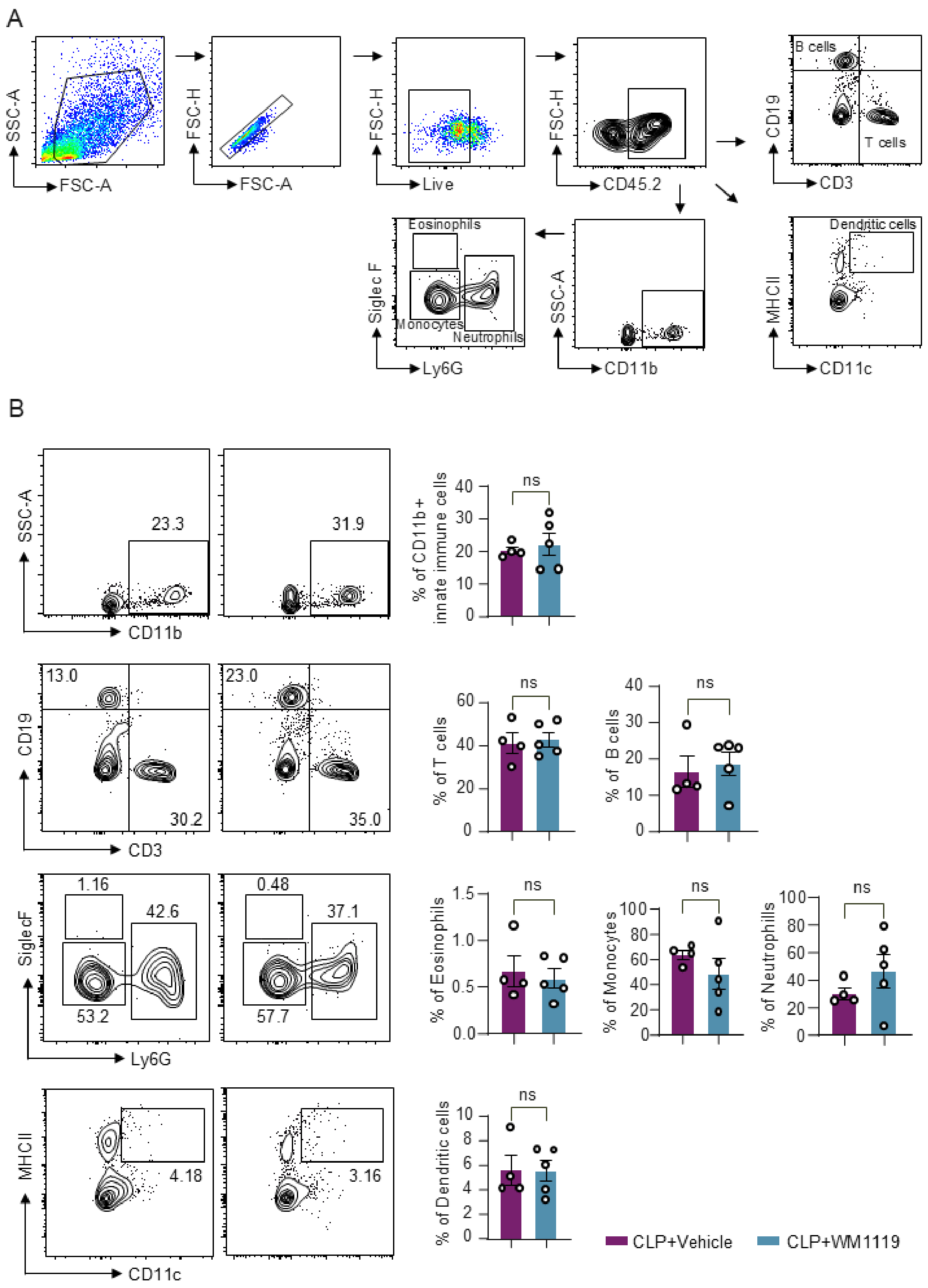

Figure S7: Effect of KAT6A inhibition on immune cell subset composition. (**A**)

Gating strategy. (**B**) The percentage of innate immune cells ($CD45^{+}CD11b^{+}$), T cells ($CD45^{+}CD3^{+}$), B cells ($CD45^{+}CD19^{+}$), eosinophils ($CD45^{+}CD11b^{+}Ly6G^{-}SiglecF^{+}$), monocytes ($CD45^{+}CD11b^{+}Ly6G^{-}SiglecF^{-}$), neutrophils ($CD45^{+}CD11b^{+}Ly6G^{+}$), dendritic cells ($CD45^{+}MHCII^{+}CD11c^{+}$) in septic lung tissues treated with WM1119 or vehicle. Data are mean ± SEM. Statistics were done by two-tailed unpaired Student's t test in panel B. ns, not significant.

A

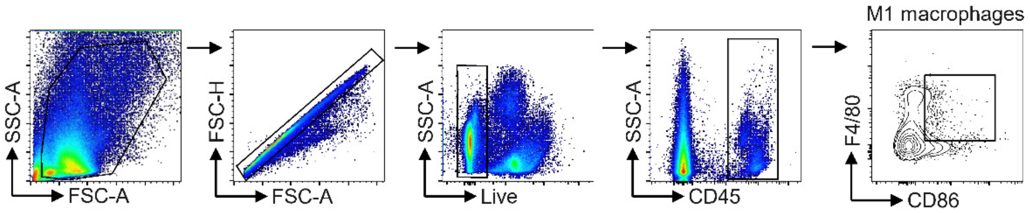

Figure S8: Gating strategy for flow cytometry analysis of macrophages in lung tissues.

Figure 1 J

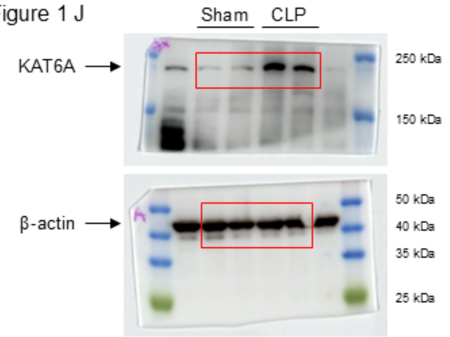

Figure 2 G

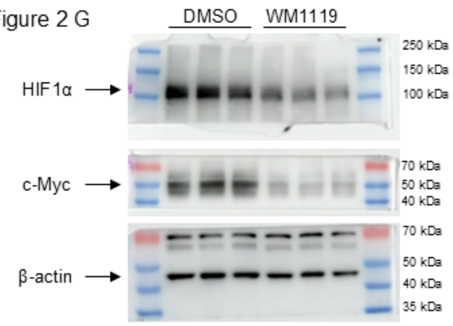

Figure 2 H

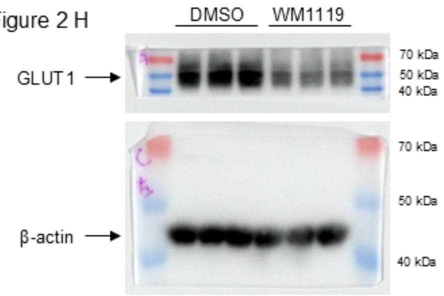

Figure 2 I

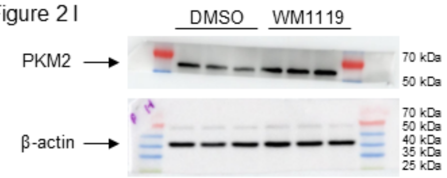

Figure 4 E

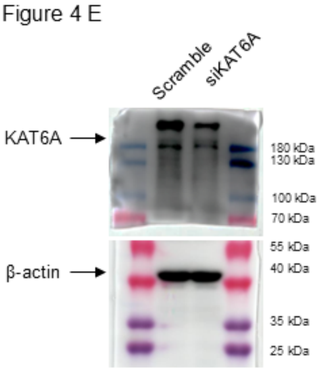

Figure 4 J

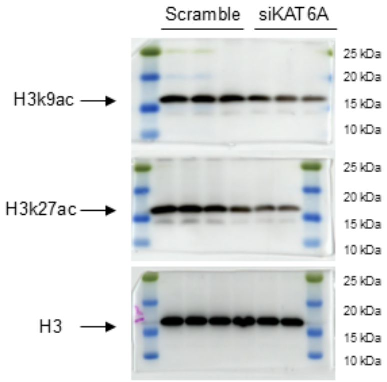

Figure 5 D

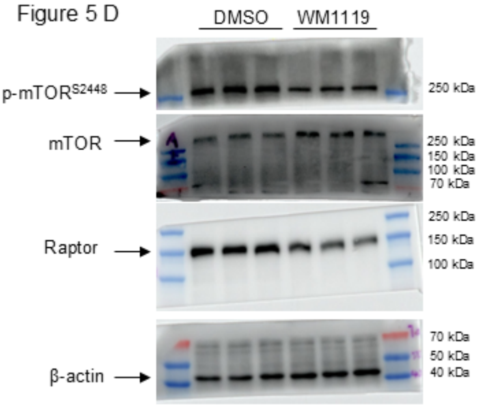

Figure 5 E

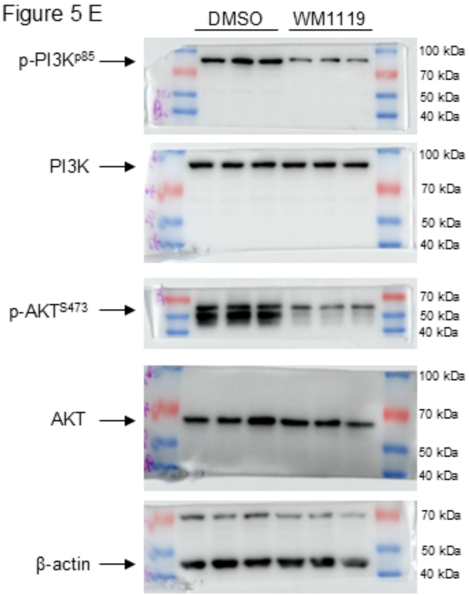

Figure S9: Uncropped gels for western blot in Figure 1, Figure 2, Figure 4 and Figure 5.

Gene	Forward primer	Reverse primer
Mouse *Kat6a*	ATGGTAAAACTCGCTAACCCG	CGTCCCGTCTTTGACGCTC
Mouse*Tnfα*	CCCTCACACTCAGATCATCTTCT	GCTACGACGTGGGCTACAG
Mouse *Nos2*	ACATCGACCCGTCCACAGTAT	CAGAGGGGTAGGCTTGTCTC
Mouse *IL-6*	GTATGAACAACGATGATGCACTTG	ATGGTACTCCAGAAGACCAGAGGA
Mouse *IL-10*	GCTGGACAACATACTGCTAACC	ATTTCCGATAAGGCTTGGCAA
Mouse *IL-1β*	CTTCAGGCAGGCAGTATCACTC	TGCAGTTGTCTAATGGGAACGT
Mouse *Aldoc*	AGAAGGAGTTGTCGGATATTGCT	TTCTCCACCCCAATTTGGCTC
Mouse *Tpi1*	CCAGGAAGTTCTTCGTTGGGG	CAAAGTCGATGTAAGCGGTGG
Mouse *Eno1*	TGCGTCCACTGGCATCTAC	CAGAGCAGGCGCAATAGTTTTA
Mouse *Aldh9a1*	GGCCGAGTGATTGCCACTT	AGGCCACTTTTCTTACTCCAGA
Mouse *Ldha*	TGTCTCCAGCAAAGACTACTGT	GACTGTACTTGACAATGTTGGGA
Mouse *Pkm*	GCCGCCTGGACATTGACTC	CCATGAGAGAAATTCAGCCGAG
Mouse *Pgk1*	ATGTCGCTTTCCAACAAGCTG	GCTCCATTGTCCAAGCAGAAT
Mouse *Pgam1*	TCTGTGCAGAAGAGAGCAATCC	CTGTCAGACCGCCATAGTGT
Mouse *Hk2*	TGATCGCCTGCTTATTCACGG	AACCGCCTAGAAATCTCCAGA
Mouse *Gapdh*	CATCACTGCCACCCAGAAGACTG	ATGCCAGTGAGCTTCCCGTTCAG
Mouse *Glut1*	CTCTGTCGGCCTCTTTGTTAAT	CCAGTTTGGAGAAGCCCATAAG
Mouse *Hif1α*	ACCTTCATCGGAAACTCCAAAG	ACTGTTAGGCTCAGGTGAACT
Mouse *c-Myc*	ATGCCCCTCAACGTGAACTTC	CGCAACATAGGATGGAGAGCA
Mouse *β-actin*	GGCTGTATTCCCCTCCATCG	CCAGTTGGTAACAATGCCATGT

Table S1: List of qPCR primer sequences
